# Identification of the distribution of human endogenous retroviruses K (HML-2) by PCR-based target enrichment sequencing

**DOI:** 10.1186/s12977-020-00519-z

**Published:** 2020-05-06

**Authors:** Bei Xue, Tiansheng Zeng, Lisha Jia, Dongsheng Yang, Stanley L. Lin, Leonardo A. Sechi, David J. Kelvin

**Affiliations:** 1 Division of Immunology, Shantou University Medical College, Shantou, China; 2 The Department of Microbiology and Immunology, Dalhousie University, Halifax, Canada; 3 Canadian Center for Vaccinology, Dalhousie University, Halifax, Canada; 4 Department of Biomedical Sciences, University of Sassari, Sassari, Italy

**Keywords:** HERV-K (HML-2), PTESHK, Genome-wide distribution, Polymorphic loci

## Abstract

**Background:**

Human endogenous retroviruses (HERVs), suspected to be transposition-defective, may reshape the transcriptional network of the human genome by regulatory elements distributed in their long terminal repeats (LTRs). HERV-K (HML-2), the most preserved group with the least number of accumulated of mutations, has been associated with aberrant gene expression in tumorigenesis and autoimmune diseases. Because of the high sequence similarity between different HERV-Ks, current methods have limitations in providing genome-wide mapping specific for individual HERV-K (HML-2) members, a major barrier in delineating HERV-K (HML-2) function.

**Results:**

In an attempt to obtain detailed distribution information of HERV-K (HML-2), we utilized a PCR-based target enrichment sequencing protocol for HERV-K (HML-2) (PTESHK) loci, which not only maps the presence of reference loci, but also identifies non-reference loci, enabling determination of the genome-wide distribution of HERV-K (HML-2) loci. Here we report on the genomic data obtained from three individuals. We identified a total of 978 loci using this method, including 30 new reference loci and 5 non-reference loci. Among the 3 individuals in our study, 14 polymorphic HERV-K (HML-2) loci were identified, and solo-LTR330 and N6p21.32 were identified as polymorphic for the first time.

**Conclusions:**

Interestingly, PTESHK provides an approach for the identification of the genome-wide distribution of HERV-K (HML-2) and can be used for the identification of polymorphic loci. Since polymorphic HERV-K (HML-2) integrations are suspected to be related to various diseases, PTESHK can supplement other emerging techniques in accessing polymorphic HERV-K (HML-2) elements in cancer and autoimmune diseases.

## Background

Human endogenous retroviruses (HERVs) are relics of ancient germ cell infection by exogenous retroviruses that became incorporated into germ line DNA [1]. HERVs are thought to have been co-opted into physiological roles in the host even though previously they have been regarded as “junk DNA”. The more direct advantage for the host of endogenization may be the protection against infection by related exogenous viruses [2]. Furthermore, HERVs have been shown to be important determinants of pluripotency in human embryonic stem cells and in the reprogramming process of induced pluripotent stem cells [3]. HERV-K (HML-2), the best evolutionarily preserved and most biologically active group among the HERVs, maintain coding competence with complete, or near-complete ORFs for all viral proteins [4, 5]. They have elevated transcripts expression, induce antibodies to viral proteins, such as gag and env, and generate virus-like particles in certain types of diseases [6, 7]. Increasing numbers of studies are now focusing on the pathogenetic mechanisms of HERV-K (HML-2) [8, 9]. One mechanism is the viral proteins produced by HERV-K (HML-2); for example, rec, np9, and env, may work as onco-proteins. Another important mechanism is the regulatory functions of LTRs, which may enable the LTRs to serve as genome-wide regulators, including alternative promoters, enhancers, polyadenylation signals, and binding sites for transcription factors [10].

HERV-K (HML-2) is the only group of HERV-K containing human-specific and polymorphic loci. Polymorphic loci that are unfixed in the modern human population suggest that they remained transcriptionally active up to very recent times in the evolutionary history of the *Homo Sapiens* species. When compared with other HERV loci, HERV-K (HML-2) have fewer accumulated mutations, indicating that they likely continue to co-evolve with the host, and they are probably still biologically active at present. Insertionally polymorphic HERV-K (HML-2) loci show different structural features, including full-length provirus integration, solo-LTR integration and unoccupied pre-integration, in different individuals [11, 12]. Therefore, polymorphic HERV-K (HML-2) integrations have the capacity to alter the expression of viral proteins as well as LTR regulation of host-genes. Several polymorphic loci have been reported to be correlated with diseases. For example, the distribution of two polymorphic HERVs, HERV-K113 and HERV-K115, have been shown to be associated with autoimmune diseases [13, 14]. A polymorphic HERV-K (HML-2) solo-LTR insertion (1p13.2) is possibly involved in lung adenocarcinoma [15]. Additionally, a polymorphic locus, in 5q14.1, integrated within RASGRF2, can disrupt host gene transcription and is associated with drug addiction [16]. Therefore, studies on the genome-wide distribution of HERV-K (HML-2) to identify polymorphic insertion loci should enable the possible identification of disease-related genes and their regulation by HERV-K (HML-2) elements.

Recently, several studies have focused on positioning of the location of HERV-K (HML-2) in the human genome (Table 1). In 2011, Subramanian et al. mined the hg19 genome and identified that more than one thousand proviral and solo-LTR HERV-K (HML-2) integrations exist in the hg19 genome [17]. Researchers focused attention on non-reference loci that do not exist in hg19. With the emergence of next generation sequencing (NGS), several data-mining tools have been established capable of evaluating the status of HERV-K (HML-2) from whole genome sequencing (WGS) data [18–21]. Dozens of non-reference and unfixed loci now complement previous HERV-K (HML-2) data. To determine the accuracy of genotyping HERV-K (HML-2) integrations, higher depth WGS data is needed; however, the cost in time and money is high, although information of the distribution of HERV-K (HML-2) makes it is possible to characterize the polymorphic nature of HERV-K (HML-2) integrations. Locus-specific PCR can target tens of loci instead of only one or two loci; however it is usually limited to throughput using Sanger sequencing [22–24]. A modified PCR technique, genome-wide amplification of proviral sequences (GAPS), established to identify polymorphic proviral loci, is also restricted by the restriction enzyme (*Vsp*I) cutting sites and sequencing method [25]. Therefore, development of an appropriate and economical method, requiring a low volume of sequencing data but capable of identifying a large number of loci, is important. Target enrichment sequencing may be an ideal approach. A specific PCR enrichment protocol targeting HERV-K (HML-2) proviruses has been successful in identifying gorilla-specific loci integrated polymorphisms [26]. A probe-based enrichment sequencing method targeting proviral and solo-LTR integrations has been reported [16, 27]. However, enrichment sequencing techniques using the Miseq platform produces fewer reads compared with Hiseq. In the present study, we established a PCR-based target enrichment sequencing of HERV-K (HML-2) (PTESHK) that both detects reference loci and identifies novel loci, which will be helpful for completing the full genomic distribution of HERV-K (HML-2) in the current reference genome. Furthermore, PTESHK is also capable of identifying polymorphic insertion loci by comparing the relative abundance of the same loci in different individuals. PTESHK could be a supplementary technique to detect disease-related polymorphic HERV-K (HML-2) loci and advance research on the contribution of HERV-K (HML-2) to human diseases.

**Table 1:** Techniques for identifying the position of HERV-K (HML-2)

Techniques	Need sequencing?	Target enrichment?	High throughput?	Detectable loci	Reference
Mining in the reference human genome (hg19)	No	No	Yes	Proviruses and solo-LTR	Subramanian et al. [17]
Locus-specific PCR	Yes, Sanger sequencing	Yes, PCR	No	Proviruses or solo-LTR	Otowa et al. [22] Moyes et al. [23] Wildschutte et al. [24]
Genome-wide amplification of proviral sequences (GAPS)	Yes, Sanger sequencing	Yes, PCR	No	Proviruses	Macfarlane and Badge [25]
Mining in whole-genome sequencing (WGS) data	Yes, WGS	No	Yes	Proviruses and solo-LTR	Wildschutte et al. [19] Wallace et al. [18] Hollway et al. [26]
Customized target enrichment method	Yes, Miseq PE300	Yes, probe-based enrichment	Yes	Proviruses and solo-LTR	Santander et al. [27] Karamitors et al. [16]
Specific PCR enrichment protocol target HML-2 proviruses	Yes, Miseq	Yes, PCR-based enrichment	Yes	Proviruses	Hollway et al. [26]
PCR-based target enrichment sequencing of HERV-K (HML-2) (PTESHK)	Yes, Hiseq X ten	Yes, PCR-based enrichment	Yes	Proviruses and solo-LTR	This study

## Results

### Collection of all known HERV-K (HML-2) loci

A clear knowledge of the integration loci and nucleotide sequences of the HERV-K (HML-2) is paramount to understanding their biological functions. Recent discoveries have catalogued full-length and partial HERV-K (HML-2) proviruses, and LTR elements lacking other HERV-K internal sequences (solo LTRs) in the human genome. In addition to the reference loci found in the GRCh37 (hg19) genome [17], dozens of non-reference (not present in the human reference genome) loci have been identified from whole genome sequencing (WGS) data using different analytic tools [19–21]. In total, there are 1063 reported loci whose insertional elements possess functional elements, including 85 reference provirus insertions, 946 reference solo-LTR insertions, 5 non-reference provirus insertions, and 27 non-reference solo-LTR insertions [17–21]. Among them, 69 have been confirmed to be polymorphic insertion (unfixed) loci [17–20, 28] and 89 seem to be fixed in Chinese populations [19]. We collected information on known HERV-K (HML-2) (Additional file 1: Table S1) for comparative analysis with data generated by target enrichment.

### PCR-based target enrichment sequencing of HERV-K (HML-2) (PTESHK)

**Fig. 1:**
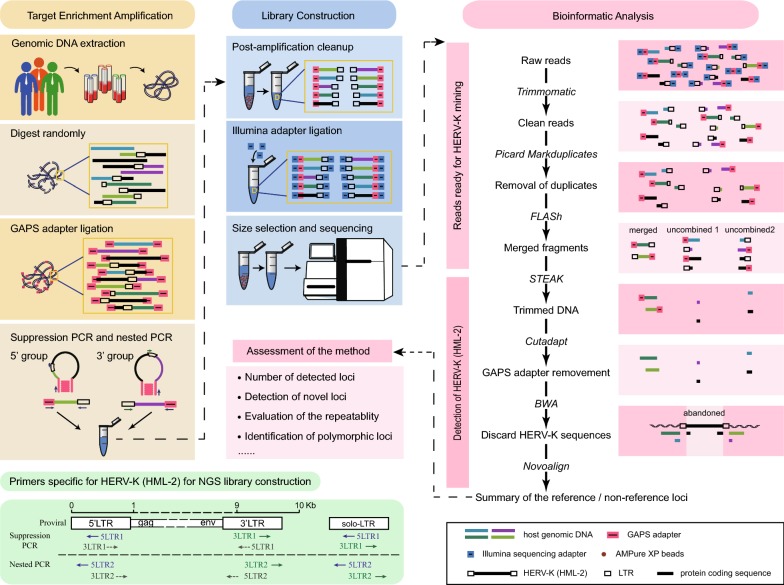
Workflow diagram of the PCR-based target enrichment sequencing of HERV-K (HML-2) (PTESHK). The workflow is divided into three phases: target enrichment amplification, library construction and bioinformatic analysis. Genomic DNA is randomly enzymatically digested at 30 °C for 5 min to form DNA fragments. Then, after end-repair and A-tailing, a GAPS adapter, to be used for the amplification, is ligated. Suppression PCR is then performed with specific primers targeting the GAPS adapters and 5′/3′ LTR sequences of HERV-K. Nested PCR with inner primers is then used to increase the amplification specificity. After amplification, PCR products of both the 5′ and 3′ ends are mixed together and cleaned with beads. Another end-repair and A-tailing is performed for the ligation of Illumina sequencing adapters, followed by size-selection. Then the library is ready for sequencing on the HiSeq X Ten platform. After sequencing, all sequencing reads are filtered by Trimmomatic to remove low-quality reads, followed by the discarding of PCR duplicates using Picard. Then paired-end reads are merged based on the overlap before filtering the chimeric reads, including LTR and flanking sequences, with STEAK. After acquisition of the trimmed flanking sequence, the GAPS adapter is cut by Cutadapt, and then those reads that mapped to HERV-K sequences are abandoned after the alignment. Last, the reference loci and non-reference loci are catalogued with help of a BED file. Then analysis is done to assess the method. In addition, a schematic of all the primers specific for HERV-K (HML-2) sequence used for NGS library construction was drawn

In this study, a PCR-based target enrichment sequencing of HERV-K (HML-2) (PTESHK) has been established to amplify and determine individual HERV-K (HML-2) element insertion sites within genomic DNA. The method takes advantage of target enrichment amplification of HERV-K (HML-2) integration sites and construction of sequencing libraries for obtaining HERV-K (HML-2) individual proviruses or solo LTR sequence linked to insertion site sequences (Fig. 1).

Target enrichment amplification of HERV-K (HML-2) is a modification of the genome-wide amplification of proviral sequences (GAPS) technique previously published [25]. There are two modifications of PTESHK over the GAPS method. One is that GAPS focuses on proviral integrations, while PTESHK is ideal for all HERV-K (HML-2) insertions, including proviral HERV-K (HML-2) insertions and solo-LTR insertions. For this purpose, degenerate primers for suppression PCR and nested PCR were redesigned to be specific for the consensus LTR nucleotide sequence, which was generated after an alignment of all reference proviral HERV-K (HML-2) sequences (Fig. 1, blue solid arrow for the 5′ group, green solid arrow for the 3′ group). The other modification is that PTESHK utilizes NGS, resulting in higher throughput than GAPS. To detect as many HERV-K (HML-2) loci as possible, an enzyme that digests the genome randomly was used to replace the restriction enzyme *VspI* and the GAPS adapter was optimized for A-T ligation (details shown in Methods and Materials). Furthermore, after the amplification, the PCR products were sequenced using an NGS platform which allowed for a greater number of sequencing reads than could achieved by Sanger sequencing used in the GAPS method.

For transposable elements and retrovirus detection in high-throughput sequencing data, we used the STEAK (Specific Transposable Element Aligner (HERV-K)) tool as the core component for pipeline analysis of our data [27]. We also adjusted STEAK to improve the accuracy of mapping each location and to reduce the number of false-positive loci. As there are two LTR sequences in the host genome for each HERV-K (HML-2) provirus, a number of LTR-gag or LTR-env containing chimeric sequences, in addition to LTR-host genome sequences, will be obtained following the amplification step (generated by primers annotated by the dotted grey arrows in Fig. 1). Flanking sequences were obtained following trimming by STEAK and alignment. Sequences that showed homology to any of the reference HERV-K (HML-2) sequences (mostly *gag* and *env* sequences) were discarded, and the remaining fragments were mapped. To ensure the accuracy of the alignment to integration loci, reads smaller than 10 nt were discarded after the removal of the GAPS adapter sequence. For the same purpose, only uniquely mapped reads were extracted for the detection of the HERV-K (HML-2) loci after alignment to hg19 by Novoalign.

### Detection capability of PTESHK

In this study, three healthy Chinese males (age range 25 to 29 years; individuals labeled as “P”, “W” and “Y”) were recruited for the sample collection to establish the utility of the PTESHK method. Three replicates (experimental replicates, denoted “1”, “2” and “3”) of each individual were performed to evaluate the repeatability of the method.

**Fig. 2:**
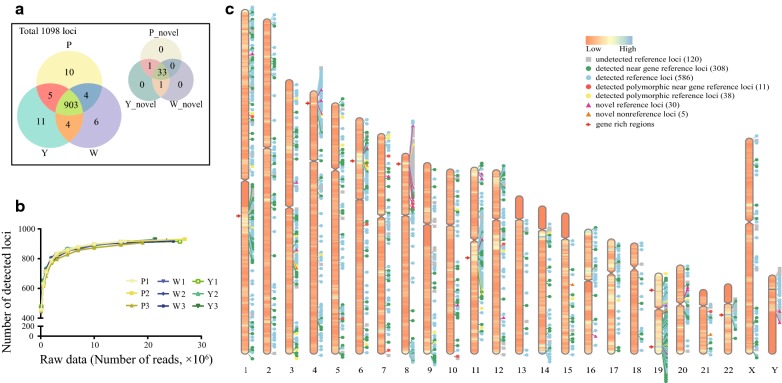
Identification and characterization of HERV-K (HML-2) loci in the human genome using PTESHK. “P”, “W” and “Y” indicate the three different individuals, each with three replicates (marked as “1”, “2”, and “3”). **a** Venn diagram exhibiting all the HERV-K (HML-2) detected with at least one single read mapped in this study. Combining the 1063 reference loci and 35 novel loci detected in this study, there were in total 1098 loci. PTESHK detected 943 reference loci and 35 novel loci. In each individual, over 900 loci could be detected in at least one repeat (957 of “P”, 951 of “W”, and 958 of “Y”). **b** Linear graph, exhibiting the number of detected HERV-K (HML-2) loci with different numbers of raw reads extracted, shows a similar variation trend for all samples. With 15 million raw reads, PTESHK was able to detect about 800 loci in every sample. **c** Genome-wide distribution of HERV-K (HML-2) loci. There were 943 detected reference loci, 120 undetected reference loci, and 35 novel loci. The detected reference loci were subdivided into near gene loci, polymorphic integrated loci, and polymorphic integrated near gene loci. Novel loci could be divided into novel reference loci and novel non-reference loci. Different groups are highlighted with different symbols in the figure. The distribution of these HERV-K (HML-2) loci shows that HERV-K (HML-2), including the 35 newly detected loci, tends to be located in gene rich regions (some highlighted by red arrows)

According to all 1063 reference loci [17, 19–21], any locus with at least one single read mapping within 10 bp of an original reference locus was considered as a detected reference locus. In all 9 samples, 943 out of 1063 reference loci were detected (Fig. 2a). To determine the number of HERV-K loci identified by PTESHK with increasing read counts, we obtained increasing numbers of reads from raw sequence data of different samples and identified the number of HERV-K (HML-2) loci. All HERV-K (HML-2) integration loci with at least a single mapped read are summarized in Fig. 2b. All 9 samples, comprised of three repeat samples from each of the three individuals, exhibited a similar trend of variation, with a peak value of about 910 detectable loci plateauing when the number of reads reached approximately 15 million.

We also discovered several novel loci using PTESHK. Following the initial analysis of our samples, many reads were found to map to sites located more than 2 kb away from the known reference loci. These loci were therefore classified as novel loci. Integrative Genomics Viewer (IGV) was then used to visualize and localize the position and determine the genomic environment of the region. Thirty solo-LTR insertion loci with a relatively high detection rate were found to be novel reference loci whose sequences could be found in hg19 but have previously not been reported (Table 2). In addition, we found 5 non-reference loci shown by the target duplicated sequence (Additional file 2: Fig. S1) whose integration sites were all within repeat elements. Specific primers were designed and used to verify these 5 loci. One of the novel loci, located on chromosome 6 (denoted as N6p21.32), could be amplified using nested PCR and had a 962 bp solo-LTR (LTR5_Hs) insertion (GenBank accession number: MN325700). Unfortunately, the remaining four novel loci were found within repeat elements and could not be amplified using nested PCR. The location within repeat elements and the low detection abundance of the 4 loci compared with N6p21.32 (visualized by IGV in Additional file 2: Fig. S1) may be the reason for our difficulties in verifying their location. In total, 35 novel loci have been discovered from examination of only three individuals, suggesting there might be additional HERV-K (HML-2) proviruses and solo LTRs that remain undetected.

**Table 2:** Novel HERV-K (HML-2) integration loci

Locus	Coordinate	Orientation	Type	Flanking region
Reference HERV-K (HML-2) integration loci				
solo-1p35.1	Chr1: 33529117-33530085	(+)	LTR5_Hs	–
solo-3p12.3	Chr3: 75586870-75587898	(+)	LTR5A	1.3 kb upstream of CTD-2023G6.3
solo-3q21.2	Chr3: 125518094-125519118	(−)	LTR5A	1.3 kb downstream of LOC105374312
solo-4p16.3	Chr4: 4076017-4077040	(+)	LTR5A	BC042823 (exon5/5)
solo-4p16.1a	Chr4: 9036143-9037177	(−)	LTR5A	–
solo-4p16.1b	Chr4: 9568938-9569963	(−)	LTR5A	AF073924 (intron 13/41)
solo-6p22.2	Chr6: 25999459-26000425	(+)	LTR5_Hs	U91328.19 (exon 2/2)
solo-7p14.3	Chr7: 30758200-30759205	(−)	LTR5B	INMT (intron 1/3)
solo-8p23.1a	Chr8: 12481601-12482613	(+)	LTR5A	LOC729732 (intron 2/5)
solo-8p23.1b	Chr8: 6984509-6985529	(−)	LTR5A	–
solo-8p23.1c	Chr8: 7957620-7958646	(−)	LTR5A	FAM85B (exon 4/4)
solo-8p12	Chr8: 29976041-29976903	(−)	LTR5B	LEPROTL1 transcript variant 2 (intron3/3)
solo-10q24.1	Chr10: 99176602-99177875	(−)	LTR5_Hs	0.6 kb upstream of LOC644215, with Alu int
solo-11q12.2	Chr11: 60480658-60481952	(−)	LTR5_Hs	MS4A8 (intron5/6), with Alu int
solo-11p15.4a	Chr11: 3566582-3567605	(+)	LTR5A	LOC101927708 (intron1/2)
solo-11p15.4b	Chr11: 4481698-4482706	(−)	LTR5B	–
solo-12q23.3	Chr12: 108220231-108221198	(−)	LTR5_Hs	KJ893228 (coding PRDM4) (intron 4/13)
solo-16q23.1	Chr16: 75848903-75850194	(−)	LTR5_Hs	with Alu int
solo-19q13.41	Chr19: 53435868-53437508	(−)	LTR5B	ZNF321P (intron1/1), with Alu int
solo-19p12	Chr19: 23459830-23461346	(−)	LTR5B	with LTR6A int
solo-20p11.21a	Chr20: 23736917-23737884	(−)	LTR5_Hs	
solo-20p11.21b	Chr20: 23674997-23675964	(−)	LTR5_Hs	
solo-Yq11.223	ChrY: 26026513-26027237	(−)	LTR5_Hs	–
solo-Yq11.23	ChrY: 27935164-27935888	(+)	LTR5_Hs	–
solo-Un212	Un_gl000212: 79707-80671	(+)	LTR5_Hs	–
solo-Un219	Un_gl000219: 31165-32127	(−)	LTR5_Hs	–
solo-Un222	Un_gl000222: 107341-108304	(−)	LTR5_Hs	–
solo-Un231	Un_gl000231: 22479-23506	(+)	LTR5A	–
solo-Un232a	Un_gl000232: 2327-3289	(+)	LTR5_Hs	–
solo-Un232b	Un_gl000232: 8571-9521	(−)	LTR5_Hs	–
Non-reference HERV-K (HML-2) integration loci				
N3q22.1	Chr3: 130166557-130166563	(+)		HERVK11-int, COL6A5 (intron 36/41)
N6p21.32	Chr6: 32643459-32643464	(+)	LTR5_Hs	L1PA10
N15q21.2	Chr15: 51650823-51650829	(+)		HERV17-int, GLDN (intron 1/9)
N19q11	Chr19: 28198583-28198588	(+)		inserted into HERV17-int
N21q22.11	Chr21: 33824215-33824220	(−)		MLT1C, EVA1c (intron 1/7)

Using PTESHK for analysis of 3 different individuals with 3 replicates of each led to the identification of 978 (out of 1098) HERV-K (HML-2) loci (Fig. 2a). Both the 978 detected loci and 120 undetected loci are summarized by their chromosome coordinates in Fig. 2c. In addition to the number of HERV-K (HML-2) proviral or solo LTR elements in each chromosome being significantly related to their gene content [17], the distribution of the HERV-K (HML-2) was also concentrated in gene-rich regions (Fig. 2c, red arrows) rather than in gene-poor regions. Among the 1063 reference loci, 348 loci (near gene loci) are located in or within 2 kb of genes [16]. Most of the 35 newly detected loci in our present study were also distributed in regions with high gene density. Remarkably, 8 of the 35 novel loci were located within introns of protein-coding genes while 9 novel loci were located internally or adjacent to non-coding transcripts (Table 2). The non-random distribution of HERV-K (HML-2) in gene-rich regions might be explained in that euchromatic areas are more easily accessible for proviral integration, also demonstrating the potential of HERV-K elements for regulating gene activities.

### Evaluation of PTESHK

**Fig. 3:**
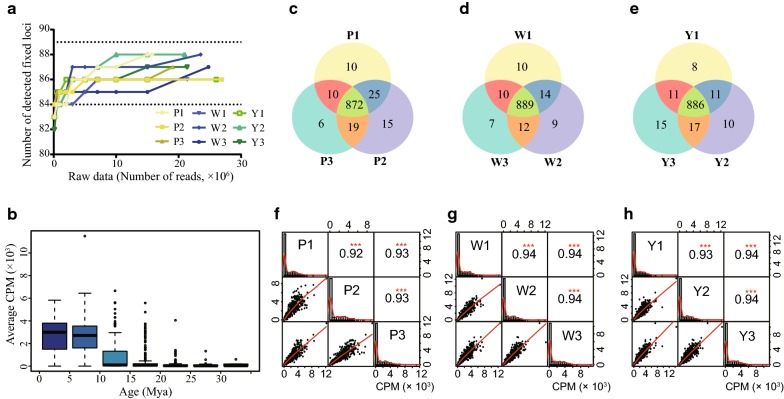
Evaluation of PTESHK. **a** Linear graph depicted the detection ability of 89 Chinese fixed loci using PTESHK. In all the samples, no less than 86 HERV-K (HML-2) fixed loci can be detected. **b** Box plot exhibiting the correlation between the average CPM of HERV-K (HML-2) loci in all 9 samples and the corresponding estimated age (Mya). As the figure shows, the more recent the HERV-K (HML-2) integration, the higher the average RPTM. These results show that it is much easier to detect recently integrated loci. **c–e** Venn diagram exhibiting the interrelationship among the detected loci of experimental replicates of different individuals. Within the three replicates of “P”, 872 loci could be detected in all the repeats, whereas in “W” and “Y”, there were 889 and 886 repeatable loci. **f–h** Correlation analysis of the relative abundance (CPM value) of the same locus in different replicates. Grids on the diagonal line of the figure represent the replicates and exhibit the distribution of the CPMs. In the upper half of the figure, the grid of intersection of the row of one sample and the column of another sample represents the Pearson correlation coefficient between the two samples and shows a significant correlation of the CPM of different samples. In the bottom half of the figure, the nearly linear correlation represents the relationship of the RPTM between every two samples

First, we evaluated the ability of PTESHK to identify HERV-K (HML-2) loci by calculating the detection of fixed HERV-K (HML-2) loci in Chinese individuals. Based on the frequency of HERV-K (HML-2) loci calculated by Wildschutte et al. among large populations, 89 loci were previously shown to be fixed in Chinese individuals with a frequency of 100% [19]. We calculated the number of detected fixed loci according to different numbers of raw reads in Fig. 3a. There are at least 86 fixed loci detected in every sample, and no less than 84 fixed loci detectable when the number of raw reads reached 1 million (Fig. 3a). Therefore, PTESHK shows good ability to detect fixed loci. Within all 9 samples in our study, there were only 5 fixed loci that were difficult to detect. Four reference solo-LTR integrations, including solo-LTR370, solo-LTR785, solo-LTR911, and solo-LTR945, are integrated in repeat elements, L1PA2, AluSx, LTR17, and beta satellite repeats, respectively. The reason these known fixed HERV-K (HML-2) integrations were more difficult to recover than others has been discussed before, which may be caused by the sensitivity of STEAK and can be resolved by lowering the tolerance or allowing for multi-location mapping [27]. A recent study has shown that another locus, located in 3q21.2, has been demonstrated to be a polymorphism [18]. Therefore, we cannot rule out the possibility that the undetectable fixed loci are polymorphic in our samples.

Next, we evaluated the repeatability of PTESHK in the total number of detected HERV-K (HML-2) among different repeats. Within the experimental replicates of the same individual, about 880 loci were detectable in all repeat experiments of each individual (872 for “P”, 889 for “W” and 886 for “Y”) (Fig. 3c–e). A Chi square test was performed to test the difference of the total number of detected loci in the different samples. Among the experimental replicates of both “W” and “Y”, there was no significant difference (*p* = 0.695 for “W” and *p* = 0.279 for “Y”). However, there appeared to be a significant difference (*p *= 0.020 < 0.05) among the 3 repeats of “P”, which may have been caused by the high disparity of the raw data (the raw data of “P2” was about 1.7-fold that of “P1”), resulting in additional loci with only a few reads mapped. To normalize the difference among the experimental replicates caused by the difference of raw data volume and to avoid possible false-positive loci, we chose to use “counts per million (CPM)” for measuring loci. Here we set a threshold of CPM ≥ 50 for filtration. The number of detected HERV-K (HML-2) insertion loci of increasing raw sequences exhibiting a similar variation trend after screening is summarized in Additional file 3: Fig. S2. When the raw data volume reached 15 million, there appeared to be little difference in the number of detected loci with the increase of the data volume (Additional file 3: Fig. S2b). Although the number of detected loci was reduced after filtering, 712 loci that were detected in at least one sample remained (Additional file 3: Fig. S2a). More than 560 loci could be detected in all repeats of each individual (566 for “P”, 563 for “W” and 567 for “Y”) (Additional file 3: Fig. S2c–e). While testing the difference of the number of detectable loci among experimental replicates using the Chi square test, all exhibited no significant difference after CPM filtering (*p* = 0.600 for “P”, *p* = 0.291 for “W” and *p* = 0.864 for “Y”).

Finally, we evaluated the repeatability of the relative abundance (CPM value) of a same locus in different repeats. While paying attention to CPM values of different loci of the same sample, differences over tens of fold could be observed; for example, the average CPM value of all the samples of solo-LTR946 (11459.45) is about 17-fold of that of solo-LTR185 (664.56). To determine whether this difference is consistent among different experimental replicates, we used CPM to calculate the correlation coefficient of the same loci among different experimental replicates to evaluate the repeatability of PTESHK. The results showed that all Pearson correlation coefficients among the different repeats were higher than 0.92, indicating that the CPMs of the same loci were significantly correlated (Fig. 3f–h). Moreover, the correlation coefficients among different samples of different individuals were also higher than 0.85, which indicates good consistency even among different individuals. If the threshold CPM was set at 50, there also seemed to be significant correlations among different repeats with a Pearson correlation coefficient of about 0.90 (Additional file 3: Fig. S2f–h).

In conclusion, with more than 900 loci detected, PTESHK exhibits good capacity for identifying HERV-K (HML-2) integration loci from a relatively small amount of data (15 million raw reads), although PTESHK has difficulty in recovering loci integrated in repetitive elements. After filtering the CPM value, PTESHK shows excellent repeatability of the number of detected HERV-K (HML-2) loci, and the relative abundance of the same loci in different replicates exhibited good repeatability.

### Detection features of PTESHK

Although the broad distribution of CPMs of different loci in the same sample is consistent, we still wanted to know the reason for this difference. Following integration into the host genome, over time HERV-K (HML-2) proviral and solo LTR sequence can independently accumulate unique mutations in each element. As the strategy for the enrichment of HERV-K (HML-2) in this study was based on PCR amplification, variants in the primer regions, resulting in differences in PCR amplification preference, may result in a bias toward more recent insertions in a given sample. Since the number of mutations increases with time, we speculated that the bias correlates to the ages of the loci. Based on LTR sequences, there are three subtypes of HERV-K (HML-2) comprising LTR5_Hs, LTR5A and LTR5B, of which LTR5_Hs is considered to be the most recently integrated family and LTR5B to be the oldest and ancestral family [17]. To investigate the relationship between LTR types (representing the integration ages) and CPMs, we performed a phylogenetic analysis of all 1080 loci (including novel loci found in this study) that possess LTR elements and have been annotated for the type of LTR, the estimated ages of the loci, the ability to be detected by PTESHK, and the CPM value of each locus. All LTRs clustered well into three groups, although LTR5B had elements that clustered in the lineages of LTR5_Hs and LTR5A (Additional file 4: Fig. S3). This phenomenon can be explained by a previous study that clustered the proviral LTRs, and concluded that LTR5B is the oldest ancestor of both LTR5A and LTR5_Hs, and therefore LTR5A and LTR5_Hs arose independently and exclusively from LTR5B [17]. With the annotation of the estimated ages of each locus, our results confirmed that LTR5_Hs performed as the youngest integration group, especially for those loci clustered within the longest internal branches and shortest terminal branches containing the majority of the polymorphic loci. The observation that nearly all the loci were detected with a much higher CPM, except for some polymorphism loci, indicates that the LTR5_Hs subgroup was more readily detected with respect to the capacity of detection and CPM values of the loci. The relationship between the estimated age of HERV-K (HML-2) loci and the average CPM value is shown in Fig. 3b, which indicates that the more recent the integration, the higher the average CPM value. This observation implies that PTESHK has a propensity for detecting recently formed integrations, especially those younger than 15 Mya. In general, it is easier for PTESHK to detect recently integrated loci, especially the LTR5_Hs subgroup, which is more likely to generate viral proteins or serve as regulatory elements for its possession of functional sequences with fewer mutations.

### Identification of polymorphic loci

**Fig. 4:**
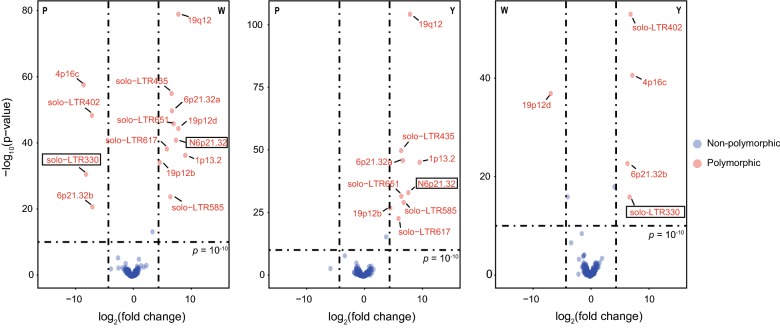
Detection of the polymorphic insertion HERV-K (HML-2) loci in three individuals. Volcano diagram exhibiting the polymorphic HERV-K (HML-2) loci between every two individuals. EdgeR was used to calculate the differential CPMs of the same loci in different individuals. If the fold change of the CPMs was higher than 20 and *p*-value less than 10^−10^, the locus was defined as polymorphic (highlighted by red). In total, there were 14 polymorphic loci with solo-LTR330 and N6p21.32 being reported for the first time as polymorphic (black frames)

Within the HERV-K (HML-2) family, there is a subset of HERV-K (HML-2) loci that are annotated as polymorphic loci raising attention from researchers. In addition to detecting the genome-wide distribution of HERV-K (HML-2) loci, we were interested in determining if PTESHK is also capable of identifying polymorphic loci between different individuals. To identify polymorphic loci, we used EdgeR to compare the differential loci between different individuals [29]. If the fold change of CPM values of the same locus in different individuals is higher than 20, and p-value less than 10^−10^, we considered the locus as polymorphic. Among the 3 individuals in our study, 14 HERV-K (HML-2) loci were determined to be polymorphic (Fig. 4). Twelve out of the 14 have previously been identified as polymorphic loci. In addition to the 12 known polymorphic loci, we found solo-LTR330 and N6p21.32 to be polymorphic (Fig. 4, black frames). Using specific primers for each polymorphic locus, 12 of the 14 loci were verified by nested-PCR. The exceptions were 12p12d and 6p21.32a, which may be caused by either their location in a repeat element, or they may represent a provirus integration in which the length of the products was too long to amplify (Additional file 5: Table S2, Additional file 6: Fig. S4).

There are 69 annotated polymorphic loci, of which 49 (71%) are detectable in this study. Besides the 12 HERV-K (HML-2) polymorphic loci, there are 37 annotated polymorphic loci denoted as nonpolymorphic and 20 denoted as undetectable loci. As the frequency of 62 out of 69 polymorphic loci have been annotated in Chinese or East Asian cohorts [18, 19] (Additional file 7: Table S3), we further evaluated the ability of PTESHK for detecting polymorphic loci by their frequency. Although there were 20 known polymorphic loci undetected in this study, we hypothesized that, rather than being undetected by PTESHK, integration at these loci was not present in our individuals. A lack of integration of these loci in Asian cohorts has been shown previously. The frequency of the 20 undetected loci is close to zero. The exception is the 12q12 locus, with a low frequency of about 0.3. There are 18 polymorphic HERV-K (HML-2) loci fixed or nearly fixed in Chinese cohorts, based on previous studies. The frequency of these loci in our 3 individuals is consistent with the observations of previous studies. For the 12 polymorphic loci detected in this study, the frequency of 8 loci is similar to that of the earlier reports. Four loci, including 6p21.32b, 19p12b, solo-LTR651 and solo-LTR585, show a relatively higher frequency in our study, which may be caused by the limitation of the number of samples. For the same reason, some loci annotated with low frequency in Asians were indicated to be fixed in our study. In general, PTESHK performs well in identifying polymorphic loci, and may be used for the detection of disease-related polymorphic HERV-K (HML-2) loci or polymorphisms in the general population of different ethnic groups.

## Discussion

HERVs and notably HERV-K insertional elements fall into two main categories: i) complete or nearly complete proviral insertions capable of synthesizing proviral RNA and in some cases producing functional viral proteins; and ii) LTR insertional elements that have the capacity for regulating expression of genes within close proximity. Integrated HERV-K proviruses and solo LTR elements have been implicated in a variety of pathological human diseases, including autoimmunity, cancer, and neurological syndromes [30–34]. Furthermore, HERV-K proviruses and solo LTRs have been shown to be important in early development [35]. Even though a large body of work has been directed at understanding the roles of HERV-K in development and disease, a complete understanding awaits a full description of HERV-K proviruses, solo LTRs, and their genomic locations. A significant problem in compiling a full list of HERV-K proviruses and solo LTRs and their genomic positions is the repetitive nature of these elements and the difficulty in assigning positions from next generation sequencing data [36]. In an attempt to generate a more complete catalogue of polymorphic and non-polymorphic HERV-K elements across ethnic groups, we modified and extended previous HERV-K discovery platforms (GAPS) in building a PCR-based target enrichment sequencing protocol for HERV-K (HML-2) (PTESHK), which can be widely applied different kinds of starting materials in limited amounts (100-150 ng genomic DNA). In addition to detecting reference loci with high efficiency, it is also capable of discovering novel HERV-K (HML-2) loci.

Compared with other techniques, PTESHK is a robust tool with several advantages for detecting HERV-K (HML-2) insertions. First, in addition to detecting proviruses integration loci [25, 26], PTESHK is also able to detect the solo-LTR integration of HERV-K (HML-2). Second, with high-throughput sequencing, hundreds of loci can be detected simultaneously. Within the 3 individuals in this study, 978 loci could be detected in at least one sample with no less than a single read mapped. PTESHK possesses a bias for the detection of the recently integrated LTR5_Hs group, which contains most of the polymorphic loci, indicative of the ability of PTESHK to detect polymorphic loci. Remarkably, 14 polymorphic loci have been identified in just 3 individuals in our study. Third, it is highly economical in that only about 15 million raw reads (about 5.4 Gb) are sufficient for characterization as compared to those studies based on WGS data with high sequencing depth (whose data volume requires around 90 Gb) [18, 19, 26]. Moreover, the analysis pipeline runs faster because of the lower volume of sequencing data. Fourth, enrichment of target sequences by PCR amplification is much easier to perform than using a biotin-streptavidin-based bead with DNA bait probes [16, 27]. Therefore, PTESHK could be helpful for determining the distribution of HERV-K (HML-2) in the human genome because hundreds of loci can be detected simultaneously with fewer raw data and excellent repeatability. Although we focused on HERV-K (HML-2), the protocol described here could be easily adapted to study different families of HERVs and possibly other retrotransposons. The application of PTESHK among different cohorts might identify polymorphic loci in specific states, for example, in different populations and within different disease types. Although PTESHK is limited in subdivided proviruses and solo-LTR HERV-K (HML-2) loci, an optimization of the suppression PCR primers could work for detecting only proviruses HERV-K (HML-2) loci [26].

In this study, we used three biological repeats and each has three experimental repeats to calculate and evaluate the repeatability of PTESHK. As shown in Fig. 3c–e, dozens of loci (ranging from 26 to 50) will be missed if studying one individual with one sample without repeats. All these loci show a similarity in that they can only be mapped with a few reads even when they are detectable. They can be clustered into two conditions. One condition is that low number of reads identified for integration groups, LTR5A and LTR5B, could be the result of difficulties in amplifying these regions because of mutations in the primer region. The other condition are those loci within repetitive elements that are more difficult to recover because of the artificial filtration of non-specific aligned reads. However, the HERV-K (HML-2) loci clustered into group LTR5_Hs, which showed high amplification efficiency, and were not integrated into repeat elements were affected less. In addition, the phenomenon of false-negative loci can be improved by increasing the data volume.

The discovery of novel loci is important for supplementing the global distribution of HERV-K (HML-2) in the reference genome. In this study, within 3 healthy individuals, 35 novel loci have been detected, including 5 non-reference loci, and 8 out of 35 loci are intronic integrations. Based on the possible regulatory functions of HERV-K (HML-2) LTRs, novel loci located in the vicinity of protein coding genes may be candidates for regulating host biological activities. The N15q21.2 locus is located in the intron of *GLDN*, a gene that encodes gliomedin involved in the formation of the nodes of Ranvier and the development of the human peripheral nervous system. As mutations in *GLDN* are responsible for lethal arthrogryposis [37], a polymorphic integration of HERV-K (HML-2) in *GLDN* could influence the expression of *GLDN* and lead to disease. Another novel locus termed N3q22.1 is located in the intron of *COL6A5*, which encodes the α5 chain of the important extracellular matrix protein collagen VI. The link between COL6A5 and atopic dermatitis is controversial [38, 39], but a mechanism involving HERV-K (HML-2) integration, such as alternative splicing or the generation of antisense RNA, might resolve this conflict. In addition, newly reported reference loci solo-7p14.3, solo-8p12, solo-11q12.2, solo-12q23.3 and solo-19q13.41 are also located within the introns of genes. Remarkably, two interesting novel loci detected in this study highlight the potential function of LTRs on alternative splicing. Solo-8p23.1c is located in the exon of FAM85B and provides a polyadenylation signal for transcript termination. So does solo-4p16.3, located in BC042823, a non-coding transcript that is not only terminated by the HERV-K (HML-2) LTR, but also initiated by an ERV1 LTR. Whether these variant transcripts influence the onset of disease remains unknown. In general, the discovery of novel loci offers new vantage points for the study of the pathogenesis of diseases.

Our ultimate purpose behind developing PTESHK is the identification of genome-wide distribution of HERV-K (HML-2) in the human genome to distinguish polymorphic integrations among different individuals or cohorts. PTESHK shows good detection ability for all the 69 previously denoted polymorphic loci with high consistency in the frequency, especially those fixed or absent in Chinese. For the 12 reported polymorphic loci also identified in the present study, their frequency is consistent with the observations of previous studies, except some with a higher frequency which may be caused by the limitation of the sample number. The ability to identify the polymorphisms in 19p12b (HERV-K113) and 1p13.2, unfixed loci that have been declared to be correlated with diseases, suggests PTESHK be applied in the detection of disease-related polymorphic loci. We also identified two new polymorphic loci (solo-LTR330 and N6p21.32) among the three individuals. The solo-LTR330 is located in the flanking sequence of exon 4 of *HLA*-*DQB1*, a gene encoding the beta 1 subunit of the human leukocyte antigen (HLA)-DQ surface receptor. HLA-DQB1 is one of the high-risk candidate genes for several diseases, such as type 1 diabetes [40] and breast cancer [41]. In accordance with previously reported bidirectional promoter activity of HERV-K (HML-2) LTRs [42], the potential presence of antisense transcripts of HLA-DQB1 initiated by solo-LTR330 might down-regulate the expression level of HLA-DQB1 transcript and modulate the transcription profile. This may provide new insight for how *HLA*-*DQB1* is a hot-spot for association with several diseases. Interestingly, we found that the pre-integration (absent) loci are always mapped by a few reads instead of no read, which results in an aberrant fold change between present and absent loci. Hence, there are some points near the cut-off lines (Fig. 4). There could be two primary reasons. First, the heterogeneity of a locus in one individual may result in an aberrant fold change. Furthermore, as some of the flanking host genome sequences exhibit high similarity, when mapping the sequencing reads to the reference, there might be false alignments. This results in a few reads mapped to the absent loci and the fold change will not be infinite. We therefore created and set a filter of fold change > 20 and p-value < 10^−10^ after testing for several scenarios for polymorphic. In this study, we detected 14 polymorphic loci. With PCR verification, 12 loci can be demonstrated with two exceptions where the loci are found within repetitive elements. Verification by PCR helps in supporting the identification of polymorphic loci detection using PTESHK.

## Conclusions

We developed PCR-based target enrichment sequencing of HERV-K (HML-2), PTESHK, to amplify chimeric LTR-host genome sequences followed by next-generation sequencing to assess the genome-wide distribution of HERV-K (HML-2) in the human genome. With this method, hundreds of HERV-K (HML-2) integrations, including both known reference loci and novel loci, could be identified with good repeatability. In addition to the ability to characterize the distribution of HERV-K (HML-2) in the human genome, PTESHK performs well for the detection of polymorphic loci that might play paramount roles in the causation of disease.

## Methods

### DNA samples and extraction

Whole blood (150–200 μl) was collected from three unrelated human volunteers (males, age ranges from 25 to 29, denoted “P”, “W” and “Y”) from Shantou, China. Up to 3 different samples (denoted “1”, “2” and “3”) from each individual were prepared for genomic DNA extraction using a TIANamp Genomic DNA Kit (TIANGEN) following the manufacturer’s protocol. The genomic DNA extracted from 3 blood drawings of one individual were mixed as a pool and used as a template for novel loci PCR validation. Informed consent was obtained at the time of collection.

### Target enrichment amplification and NGS library construction

After optimization of the amplification reactions and library construction steps (data not shown), the detailed procedures are as follows. Target enrichment amplification was a modification of the GAPS method [25] and performed using a KAPA HyperPlus Kit (Roche), and all reactions were prepared on ice. Genomic DNA (100–150 ng) was digested randomly with KAPA Frag enzyme at 30 °C for 5 min using a pre-cooled thermocycler. After enzymatic fragmentation was performed, the fragments were subjected to end repair and A-tailing and incubated at 65 °C for 30 min. Then the GAPS linker (15 μM) was ligated to the fragments at 20 °C for 15 min according to the instructions of the manufacturer. The GAPS linker was prepared by mixing an equal volume of KNG-A1 and KNG-A2 (Additional file 8: Table S4) and annealing at 65 °C for 20 min followed by cooling to room temperature at a rate of 1 °C every 15 s. After the ligation, a post-ligation cleanup was performed immediately with AMPure XP beads (Beckman) to remove unligated linker or linker-dimer molecules.

Suppression PCR was carried out using Premix Taq™ (Takara) in a final volume of 20 μl containing 3 ng of ligated genomic DNA and 1.25 μM of each primer. One of the primers was designed based on the linker sequence (RBX4), and the other on the HERV-K LTR sequence (consensus sequence after an alignment of all the reported HERV-K (HML-2) provirus sequences) (5LTR1 for 5′ LTR end and 3LTR1 for 3′LTR end) (Fig. 1, Additional file 8: Table S4). PCR was performed as follows: 96 °C for 1 min; 25 cycles of 96 °C for 30 s, 60 °C for 2 min, and a final extension step at 72 °C for 10 min. Nested PCR under the same conditions was then performed, with a 1:50 dilution of PCR products, with inner primers specific to the sequence of the primary PCR product (5LTR2 for 5′ LTR, 3LTR2 for 3′ LTR and RBY1 for the linker, Additional file 8: Table S4) in a final volume of 50 μl.

After the secondary PCR, the products of both 5′LTR and 3′LTR from the same sample were combined to form a 100 μl mix for a post-amplification cleanup with AMPure XP beads (Beckman) to get a 50 μl eluate of the products. The products were subjected to an end repair and A-tailing step as described above, followed by a ligation of the specific sequencing adapter (SeqCap Adapter Kit, Roche) for Illumina sequencing. After the ligation, a post-ligation cleanup and a size selection step were performed immediately for the selection of library molecules (inclusive of adapter) in the range of 250–450 bp and readied for HiSeq X Ten PE150 sequencing. To avoid errors from sequencing, all libraries were performed in one lane. The raw data of each library ranged from 5G to 10G.

### Bioinformatics analysis

Paired-end reads were trimmed using the Trimmomatic program to remove low-quality reads [43]. Picard MarkDuplicates (http://broadinstitute.github.io/picard) was used to filter the PCR duplicates out of the clean data before being merged using FLASH (Fast Length Adjustment of Short Reads) [44]. Both merged reads and single reads were submitted to STEAK [27] for HERV-K mining in the unpaired mode with a bait length of 20 bp (beginning and end of the HERV-K LTR) and a percent identity of 90. The trimmed reads were then treated with Cutadapt [45] to remove the partial GAPS linker sequences and reads shorter than 10 bp after the trim were abandoned. Then the remaining reads were aligned to all HERV-K (HML-2) sequences in GRCh37 (hg19), and all mapped reads were removed by SAMtools [46] to avoid HERV-K sequence disturbance. Novoalign (http://novocraft.com) was chosen to map reads back to the host genome hg19, and those reads uniquely mapped could be used for the detection of reference or novel HERV-K loci with BEDTools [47]. The list of known integration sites, a reference file for BEDTools, is made up of all 1063 loci that have been previously reported and is summarized in Additional file 1: Table S1. Only those fragments mapping within 10 nt of the reference loci were calculated. IGV [48] could be used for visualization and confirmation of the results.

### Statistical analysis of the method

All loci within the 1098 loci that had at least one read mapped were accepted as positive loci. Different numbers of reads (100,000, 500,000, 1,000,000, 2,000,000, 3,000,000, 5,000,000, 7,000,000, 10,000,000 and 15,000,000) were extracted from the raw data of different samples (P1, P2, P3, W1, W2, W3, Y1, Y2 and Y3) for bioinformatics analysis. The value of the CPM of every detected locus was calculated to represent the relative abundance of the locus, and it was used for the evaluation of the difference of one locus among different samples.

Statistical analysis was conducted using R software (version 3.5.0). The distribution of HERV-K (HML-2) integration loci in the hg19 genome was exhibited by RIdeogram. The Chi square test was used to determine whether there was a significant difference among the number of HERV-K loci detected in different replicates of one individual. The Pearson correlation coefficient was used to compare the correlation of CPM of the same loci among different replicates, and the results were visualized by Performance Analytics. EdgeR was used to compare the differential loci among different individuals to determine polymorphic or non-polymorphic loci. Changes of the CPM value of the loci considered as polymorphic between two individuals were 20-fold or greater with a *p*-value ≤ 10^−10^.

### New loci validation and sequencing

Primers specific for the novel non-reference loci detected by the NGS data were designed for PCR validation. PCR was carried out using Premix Taq™ (Takara) following the manufacture’s protocol. PCR products were separated on a 1.5% agarose gel, and then purified with a Universal DNA Purification kit (TIANGEN). The purified PCR products were directly ligated into the pEASY-T3 Cloning Vector (TransGen Biotech), then transformed into competent *E. coli*. Positive recombinants were identified by blue-white color selection and sent for Sanger sequencing.

### Phylogenetic analysis

The nucleotide sequences of all 1080 HERV-K (HML-2) loci that contained an LTR, including reference loci and novel loci, were selected for the construction of a neighbor-joining tree. For those provirus insertions with two LTR elements, the 5′ LTR sequence was chosen first and if it was 5′ truncated, then the 3′ LTR sequence was used. All sequences were downloaded from NCBI (National Center for Biotechnology Information, https://www.ncbi.nlm.nih.gov/). Nucleotide sequences were aligned using ClustalW [49] and then used to generate the tree with MEGA7 [50] using 5000 bootstraps and the pair-wise deletion option. The NJ tree was visualized by the online tool iTOL (Interactive Tree Of Life, http://itol.embl.de/) together with the annotation information obtained by early statistics analysis.

## Supplementary information

**Additional file 1: Table S1.** All the reported HERV-K (HML-2) loci.

**Additional file 2: Fig. S1.** Visualization of the reads mapped to non-reference loci.

**Additional file 3: Fig. S2.** Identification of HERV-K (HML-2) loci after filtering using a CPM ≥ 50. (a) Venn diagram exhibiting the interrelationship among the detected loci of different individuals. There are still 712 loci detectable after the filter. (b) Linear graph exhibiting the different number of raw reads that were extracted to calculate the number of detectable loci. (c-e) Venn diagram exhibiting the interrelationship among the detected loci of experimental replicates of different individuals. Within the 3 replicates of “P”, 566 loci could be detected in all the repeats, while in “W” and “Y”, there were 563 and 567 loci. (f–h) Correlation analysis of the relative abundance (CPM values) of the same loci in different samples (as in Fig. 3f–h).

**Additional file 4: Fig. S3.** Phylogenetic analysis of all LTR nucleotide sequences of HERV-K (HML-2) detected by PTESHK. Phylogenetic analysis of all HERV-K (HML-2) possessing LTR sequences (provirus insertions that contained two LTRs only the 5′LTR was selected for alignment, if the 5′LTR was truncated, the 3′LTR was used). The tree was constructed by the neighbor-joining method using 5,000 bootstraps and the pair-wise deletion option. The NJ tree was well-clustered and annotated by the estimated age of every locus, the subtype of LTR (LTR5_Hs, LTR5A, and LTR5B), polymorphic insertion loci, the ability to be detected by PTESHK, and the CPM value. The results show good detection of known HERV-K (HML-2) loci, especially the LTR5_Hs type, which clustered within the longest internal branches and shortest terminal branches.

**Additional file 5: Table S2.** Nucleotide sequences for PCR verification of polymorphic loci.

**Additional file 6: Fig. S4.** Verification of polymorphic loci. Selected polymorphic loci detected by PTESHK were verified using specific primers (Additional file 5: Table S2) and separated on a 1.5% agarose gel. Primer pairs F1/R1 were used for the primary PCR performed as follows: 95 °C for 3 min; 30 cycles of 95 °C for 30 s, 52 °C for 30 s, 72 °C for 1 min per kb of the product, and a final extension step at 72 °C for 10 min. Primer pairs F2/R2 were used for the nested PCR, except 4 loci using 5LTR2 as one of the nested PCR primers (Additional file 5: Table S2). The PCR procedure was performed as follows: 95 °C for 3 min; 6 cycles of 95 °C for 30 s, 60 °C for 30 s, decreasing of 1 °C every cycle, 72 °C for 1 min per kb of the product; 30 cycles of 95 °C for 30 s, 58 °C for 30 s, 72 °C for 1 min per kb of the product, and a final extension step at 72 °C for 10 min. Among all 14 polymorphic loci, 12 loci could be verified, except for 12p12d and 6p21.32a, which may be caused by either their location in a repeat element or a provirus integration where the length of the products were too long to amplify. For 6p21.32b and 19p12b, the results were partly confirmed, as 6p21.32b of Y and 19p12b of W were not amplified. This may be caused by the difference in DNA or experimental error.

**Additional file 7: Table S3.** Prevalence of the polymorphic loci.

**Additional file 8: Table S4.** Nucleotide sequences for NGS library construction.

## Data Availability

The datasets generated and/or analysed during the current study are available in the NCBI BioProject database (http://www.ncbi.nlm.nih.gov/bioproject/) under accession number PRJNA556855.

## Abbreviations

HERV: Human endogenous retrovirus
LTR: Long terminal repeat
PTESHK: PCR-based target enrichment sequencing of HERV-K (HML-2)
WGS: Whole genome sequencing
NGS: Next generation sequencing
GAPS: Genome-wide amplification of proviral sequences
STEAK: Specific transposable element aligner (HERV-K)
IGV: Integrative genomics viewer
CPM: Counts per million
HLA: Human leukocyte antigen
FLASH: Fast length adjustment of short reads
iTOL: Interactive tree of life

## References

[1] Gifford R, Tristem M (2003). **T**he evolution, distribution and diversity of endogenous retroviruses. Virus Genes.

[2] Blanco-Melo D, Gifford RJ, Bieniasz PD (2017). Co-option of an endogenous retrovirus envelope for host defense in hominid ancestors. Elife.

[3] Grow EJ, Flynn RA, Chavez SL, Bayless NL, Wossidlo M, Wesche DJ, Martin L, Ware CB, Blish CA, Chang HY (2015). Intrinsic retroviral reactivation in human preimplantation embryos and pluripotent cells. Nature.

[4] Bannert N, Kurth R (2006). The evolutionary dynamics of human endogenous retroviral families. Annu Rev Genomics Hum Genet.

[5] Hohn O, Hanke K, Bannert N (2013). HERV-K(HML-2), the best preserved family of HERVs: endogenization, expression, and implications in health and disease. Front Oncol.

[6] Matteucci C, Balestrieri E, Argaw-Denboba A, Sinibaldi-Vallebona P (2018). Human endogenous retroviruses role in cancer cell stemness. Semin Cancer Biol.

[7] Manghera M, Ferguson J, Douville R (2014). Endogenous retrovirus-K and nervous system diseases. Current Neurol Neurosci Rep.

[8] Hanke K, Hohn O, Bannert N (2016). HERV-K(HML-2), a seemingly silent subtenant—but still waters run deep. Apmis Acta Pathologica Microbiologica Et Immunologica Scandinavica.

[9] Garcia-Montojo M (2018). Doucet-O’Hare T, Henderson L, Nath A: human endogenous retrovirus-K (HML-2): a comprehensive review. Crit Rev Microbiol.

[10] Buzdin AA, Prassolov V, Garazha AV (2017). Friends–enemies: endogenous retroviruses are major transcriptional regulators of human DNA. Front Chem.

[11] Hughes JF, Coffin JM (2004). Human endogenous retrovirus K solo-LTR formation and insertional polymorphisms: implications for human and viral evolution. Proc Natl Acad Sci USA.

[12] Moyes D, Griffiths DJ, Venables PJ (2007). Insertional polymorphisms: a new lease of life for endogenous retroviruses in human disease. Trends Genet.

[13] Moyes DL, Martin A, Sawcer S, Temperton N, Worthington J, Griffiths DJ, Venables PJ (2005). The distribution of the endogenous retroviruses HERV-K113 and HERV-K115 in health and disease. Genomics.

[14] Krzysztalowska-Wawrzyniak M, Ostanek M, Clark J, Binczak-Kuleta A, Ostanek L, Kaczmarczyk M, Loniewska B, Wyrwicz LS, Brzosko M, Ciechanowicz A (2011). The distribution of human endogenous retrovirus K-113 in health and autoimmune diseases in Poland. Rheumatology.

[15] Tomoaki K, Hong T, Kazuya S, Hidetaka Y, Hiroki M, Kazuhito F, Nobuya K, Masaya S, Masayuki T, Hiroshi N (2013). Identification and association study with lung cancer for novel insertion polymorphisms of human endogenous retrovirus. Carcinogenesis.

[16] Karamitros T, Hurst T, Marchi E, Karamichali E, Georgopoulou U, Mentis A, Riepsaame J, Lin A, Paraskevis D, Hatzakis A (2018). Human endogenous retrovirus-K HML-2 integration within RASGRF2 is associated with intravenous drug abuse and modulates transcription in a cell-line model. Proc Natl Acad Sci.

[17] Subramanian RP, Wildschutte JH, Russo C, Coffin JM (2011). Identification, characterization, and comparative genomic distribution of the HERV-K (HML-2) group of human endogenous retroviruses. Retrovirology.

[18] Wallace AD, Wendt GA, Barcellos LF, Smith AJD, Francis SS (2018). To ERV is human: a phenotype-wide scan linking polymorphic human endogenous retrovirus-K insertions to complex phenotypes. Front Genetics.

[19] Wildschutte JH, Williams ZH, Montesion M, Subramanian RP, Kidd JM, Coffin JM (2016). Discovery of unfixed endogenous retrovirus insertions in diverse human populations. Proc Natl Acad Sci USA.

[20] Emanuele M, Alex K, Gkikas M, Robert B (2014). Unfixed endogenous retroviral insertions in the human population. J Virol.

[21] Lee E, Iskow R, Yang L, Gokcumen O, Haseley P, Iii LJL, Lohr JG, Harris CC, Li D, Wilson RK (2012). Landscape of somatic retrotransposition in human cancers. Science.

[22] Otowa T, Tochigi M, Rogers M, Umekage T, Kato N, Sasaki T (2006). Insertional polymorphism of endogenous retrovirus HERV-K115 in schizophrenia. Neurosci Lett.

[23] Moyes D, Goris A, Ban M, Compston A, Griffiths D, Sawcer S, Venables P (2008). HERV-K113 is not associated with multiple sclerosis in a large family-based study. AIDS Res Hum Retroviruses.

[24] Wildschutte JH, Ram D, Subramanian R, Stevens VL, Coffin JM (2014). The distribution of insertionally polymorphic endogenous retroviruses in breast cancer patients and cancer-free controls. Retrovirology.

[25] Macfarlane CM, Badge RM (2015). Genome-wide amplification of proviral sequences reveals new polymorphic HERV-K(HML-2) proviruses in humans and chimpanzees that are absent from genome assemblies. Retrovirology.

[26] Holloway JR, Williams ZH, Freeman MM, Bulow U, Coffin JM (2019). Gorillas have been infected with the HERV-K (HML-2) endogenous retrovirus much more recently than humans and chimpanzees. Proc Natl Acad Sci.

[27] Santander CG, Gambron P, Marchi E, Karamitros T, Katzourakis A, Magiorkinis G (2017). STEAK: a specific tool for transposable elements and retrovirus detection in high-throughput sequencing data. Virus Evol.

[28] Thomas J, Perron H, Feschotte C (2018). Variation in proviral content among human genomes mediated by LTR recombination. Mobile DNA.

[29] Mark DR, Davis JM, Gordon KS (2010). edgeR: a Bioconductor package for differential expression analysis of digital gene expression data. Bioinformatics.

[30] Freimanis G, Hooley P, Davari E, Ali HA, Veitch A, Rylance PB, Alawi A, Axford J, Nevill A, Murray PG (2010). A role for human endogenous retrovirus-K (HML-2) in rheumatoid arthritis: investigating mechanisms of pathogenesis. Clin Exp Immunol.

[31] Nexø BA, Villesen P, Nissen KK, Lindegaard HM, Rossing P, Petersen T, Tarnow L, Hansen B, Lorenzen T, Hørslev-Petersen K (2016). Are human endogenous retroviruses triggers of autoimmune diseases? Unveiling associations of three diseases and viral loci. Immunol Res.

[32] Katoh I, Kurata S (2013). Association of endogenous retroviruses and long terminal repeats with human disorders. Front Oncol.

[33] Downey RF, Sullivan FJ, Feng WJ, Stefan A, Giles FJ, Glynn SA (2015). Human endogenous retrovirus K and cancer: innocent bystander or tumorigenic accomplice?. Int J Cancer.

[34] Wenxue L, Myoung-Hwa L, Lisa H, Richa T, Muzna B, Joseph S, Emilie C, Hoffman DA, Gloria VG, Kory J (2015). Human endogenous retrovirus-K contributes to motor neuron disease. Sci Transl Med.

[35] Fuchs NV, Loewer S, Daley GQ, Izsvák Z, Löwer J, Löwer R (2013). Human endogenous retrovirus K (HML-2) RNA and protein expression is a marker for human embryonic and induced pluripotent stem cells. Retrovirology.

[36] Li W, Lin L, Malhotra R, Yang L, Acharya R, Poss M (2019). A computational framework to assess genome-wide distribution of polymorphic human endogenous retrovirus-K In human populations. PLoS Comput Biol.

[37] Maluenda J, Manso C, Quevarec L, Vivanti A, Marguet F, Gonzales M, Guimiot F, Petit F, Toutain A, Whalen S (2016). Mutations in GLDN, encoding gliomedin, a critical component of the nodes of ranvier, are responsible for lethal arthrogryposis. Am J Human Genet.

[38] Söderhäll C, Marenholz I, Kerscher T, Rüschendorf F, Esparza-Gordillo J, Worm M, Gruber C, Mayr G, Albrecht M, Rohde K (2007). Variants in a novel epidermal collagen gene (COL29A1) are associated with atopic dermatitis. PLoS Biol.

[39] Esparza-Gordillo J, Weidinger S, Fölster-Holst R, Bauerfeind A, Ruschendorf F, Patone G, Rohde K, Marenholz I, Schulz F, Kerscher T (2009). A common variant on chromosome 11q13 is associated with atopic dermatitis. Nat Genet.

[40] Erlich H, Valdes AM, Noble J, Carlson JA, Varney M, Concannon P, Mychaleckyj JC, Todd JA, Bonella P, Fear AL (2008). HLA DR-DQ haplotypes and genotypes and type 1 diabetes risk. Diabetes.

[41] Manar Fayiz A, Reem Qasem T, Mahmoud SAH (2013). Negative association of the HLA-DQB1*02 allele with breast cancer development among Jordanians. Asian Pac J Cancer Prev.

[42] Domansky AN, Kopantzev EP, Snezhkov EV, Lebedev YB, Leib-Mosch C, Sverdlov ED (2000). Solitary HERV-K LTRs possess bi-directional promoter activity and contain a negative regulatory element in the U5 region. FEBS Lett.

[43] Bolger AM, Marc L, Bjoern U (2014). Trimmomatic: a flexible trimmer for Illumina sequence data. Bioinformatics.

[44] Tanja M, Salzberg SL (2011). FLASH: fast length adjustment of short reads to improve genome assemblies. Bioinformatics.

[45] Martin M (2011). Cutadapt removes adapter sequences from high-throughput sequencing reads. EMBnet journal.

[46] Li H, Handsaker B, Wysoker A, Fennell T, Ruan J, Homer N, Marth G, Abecasis G, Durbin R (2009). The sequence alignment/Map (SAM) format and SAMtools. Bioinformatics.

[47] Quinlan AR, Hall IM (2010). BEDTools: a flexible suite of utilities for comparing genomic features. Bioinformatics.

[48] Robinson JT, Thorvaldsdóttir H, Winckler W, Guttman M, Lander ES, Getz G, Mesirov JP (2011). Integrative genomics viewer. Nat Biotechnol.

[49] Larkin MA, Blackshields G, Brown NP, Chenna R, McGettigan PA, McWilliam H, Valentin F, Wallace IM, Wilm A, Lopez R (2007). Clustal W and Clustal X version 2.0. Bioinformatics.

[50] Kumar S, Stecher G, Tamura K (2016). MEGA7: molecular evolutionary genetics analysis version 7.0 for bigger datasets. Mol Biol Evol.

