# Supplementary figures and images for: Identification of the distribution of human endogenous retroviruses K (HML-2) by PCR-based target enrichment sequencing
Source: Retrovirology. 2020 May 6;17:10. doi: 10.1186/s12977-020-00519-z (PMC7201656; doi:10.1186/s12977-020-00519-z)

a: Chr3:130166557-130166563 (+)

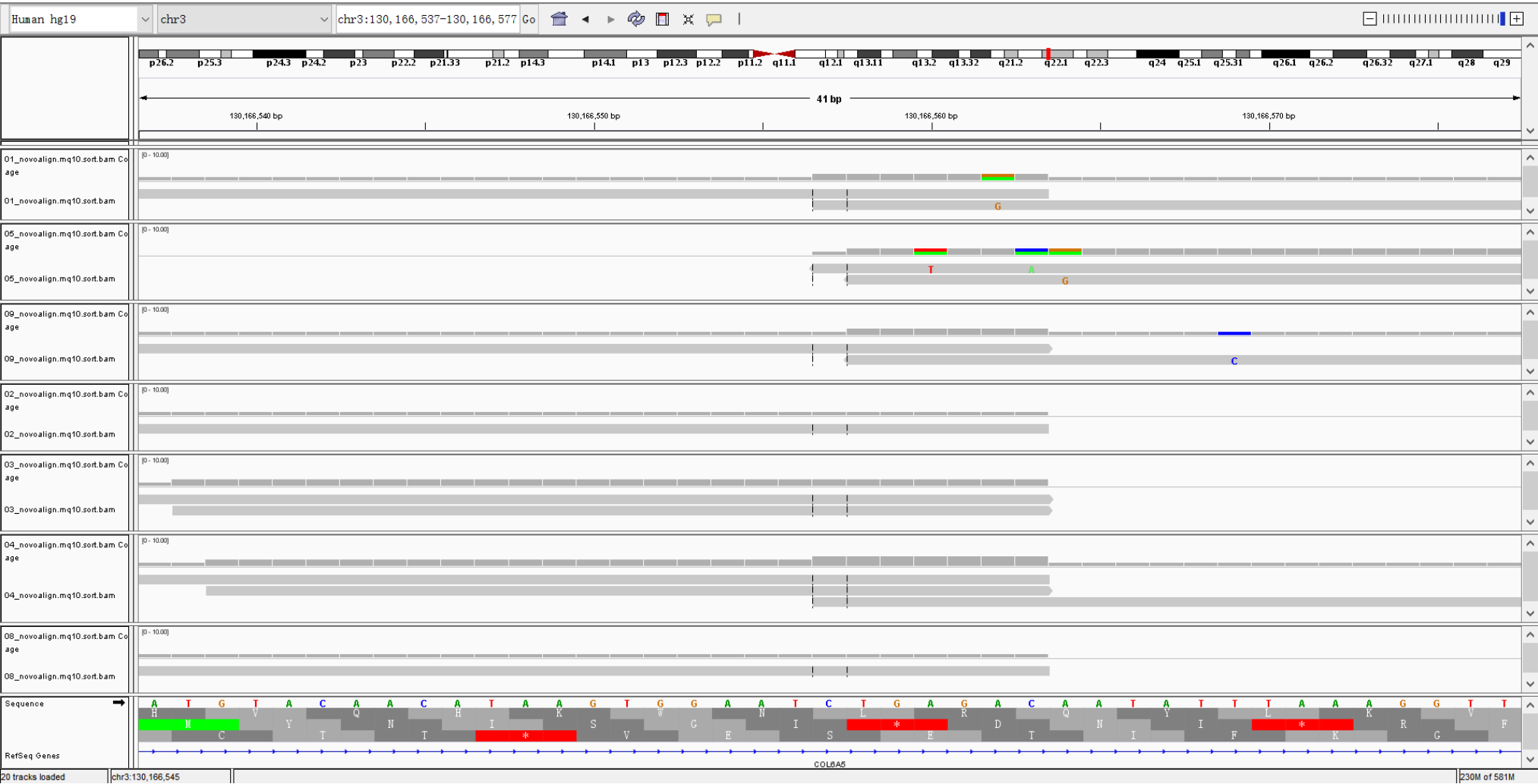

b: Chr6:32643459-32643464 (+)

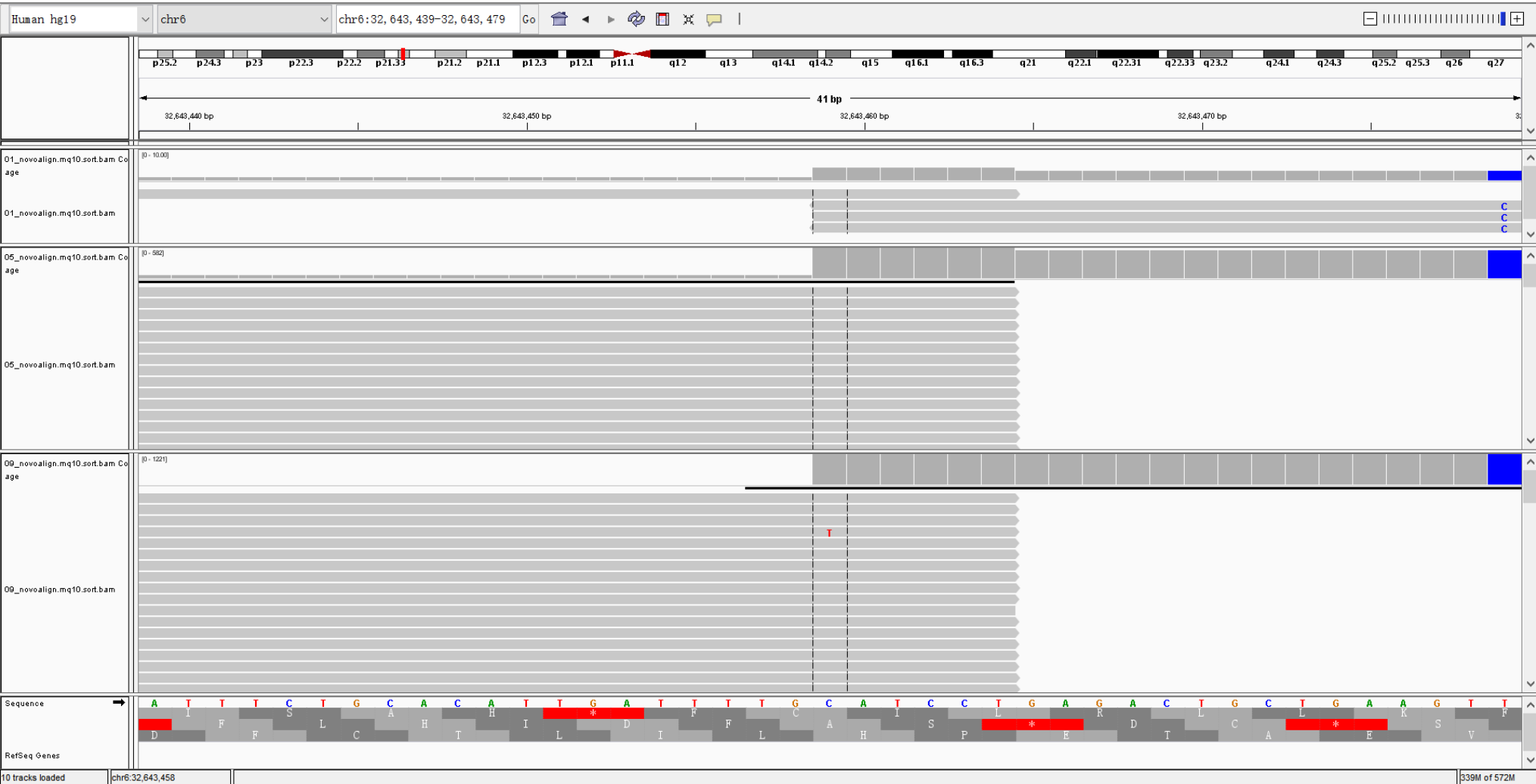

c: Chr15:51650823-51650829 (+)

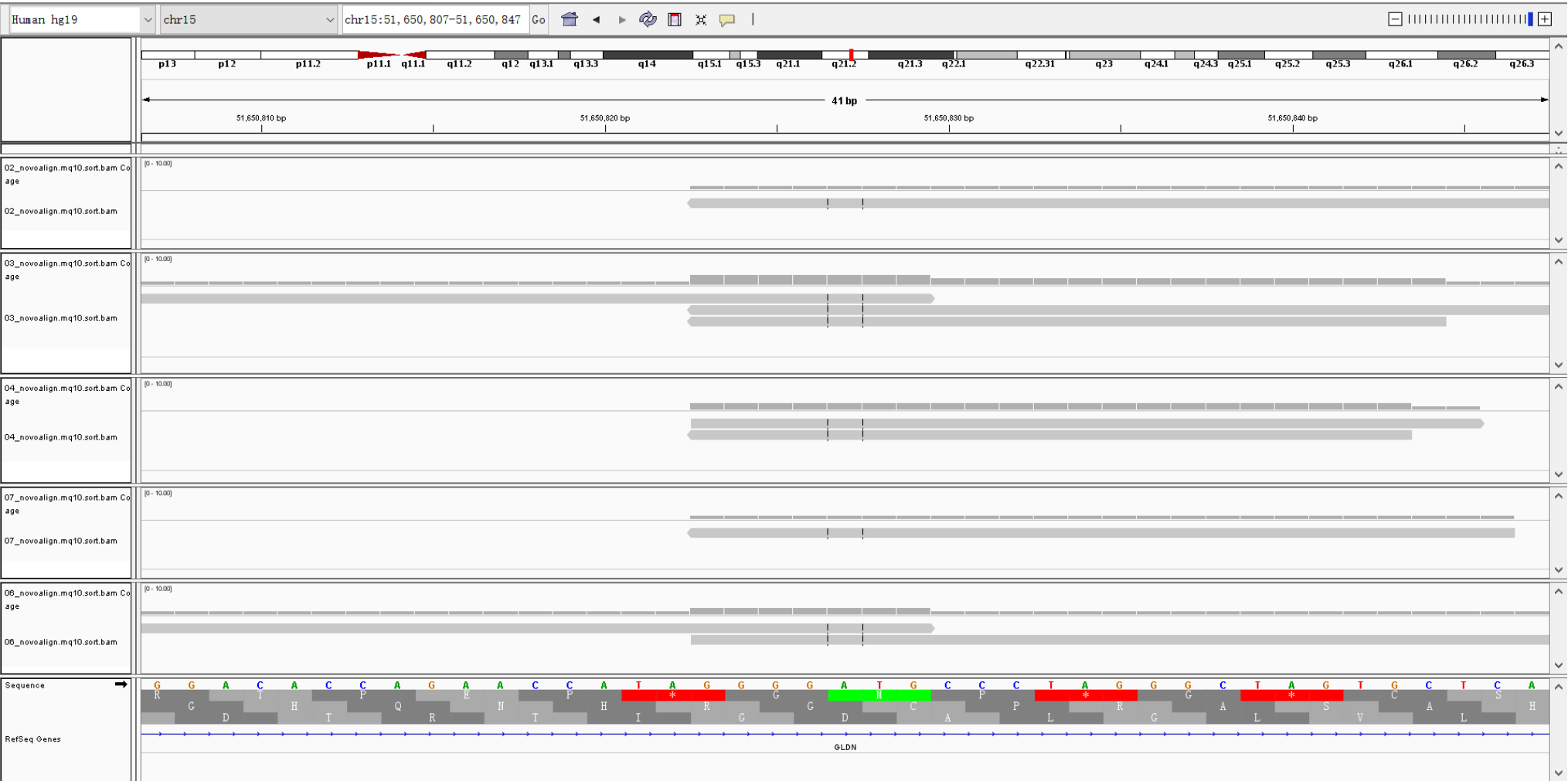

d: Chr19:28198583-28198588 (+)

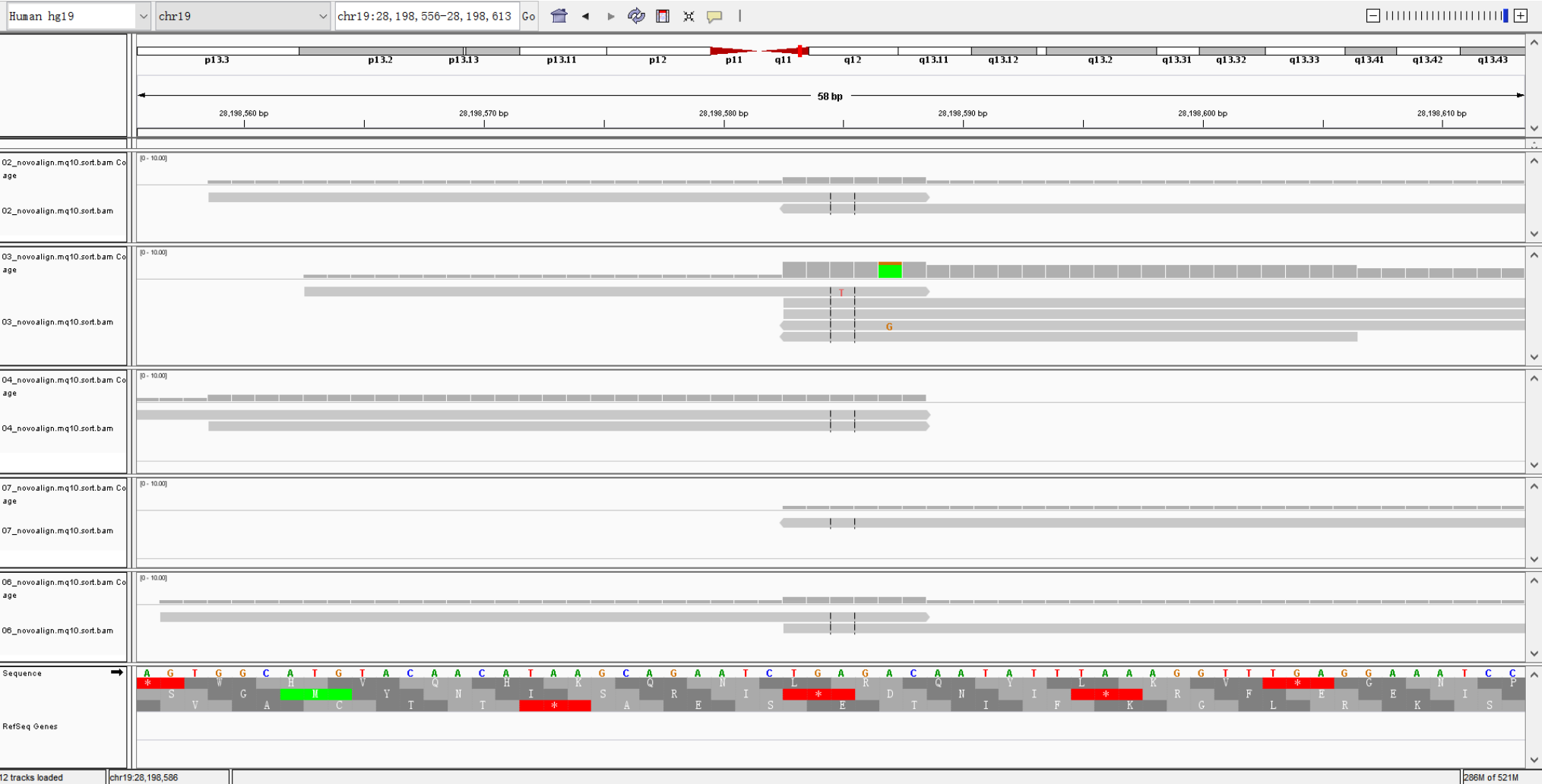

e: Chr21:33824215-33824220 (-)

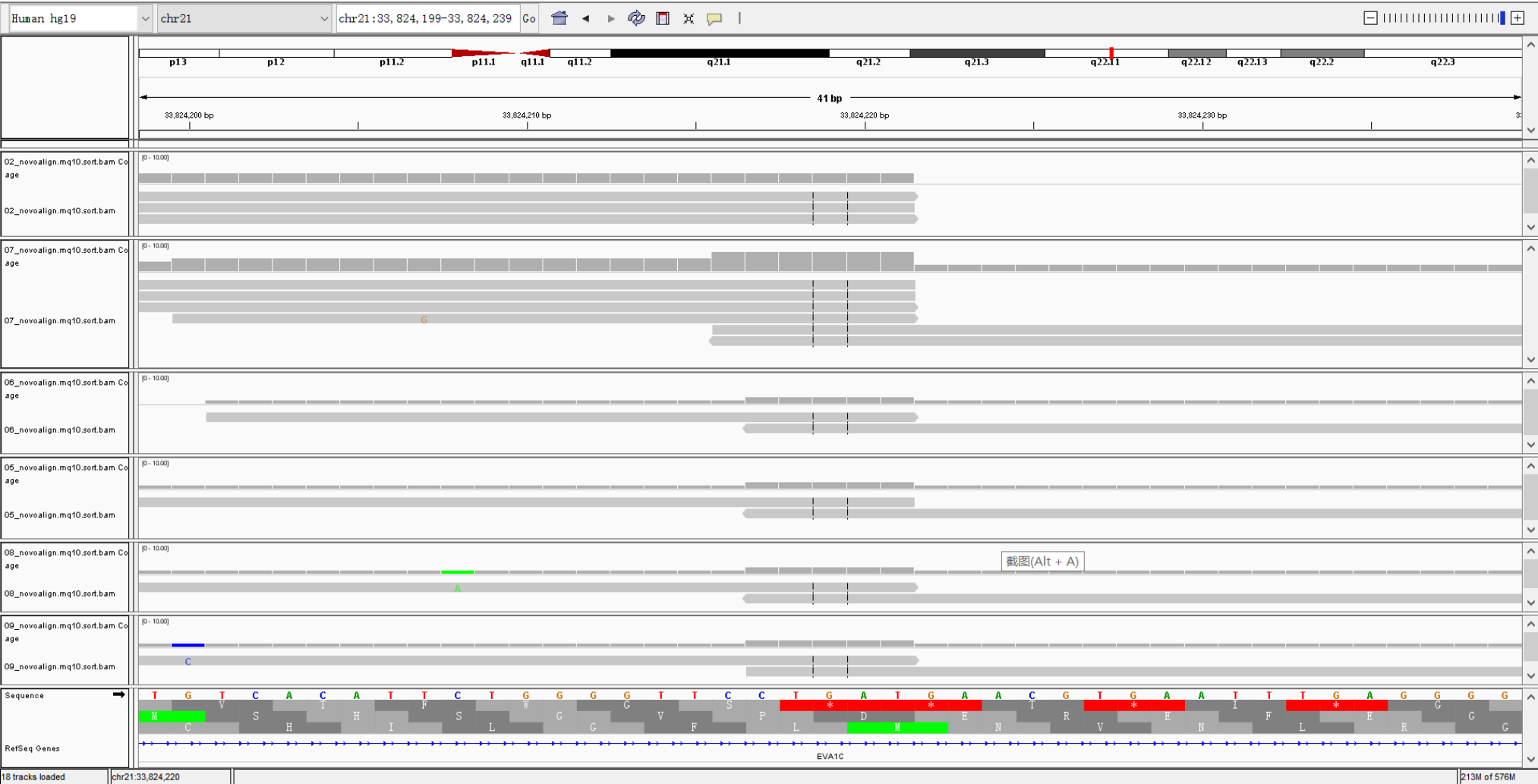

Supplement: Supplementary file 2 — Additional file 2: Fig. S1. Visualization of the reads mapped to non-reference loci. [file 12977_2020_519_MOESM2_ESM.pdf]

a

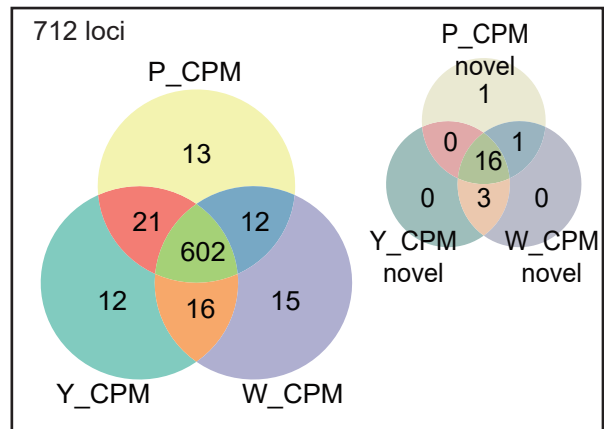

c

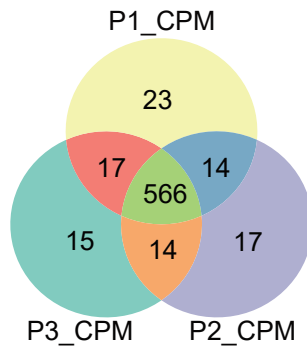

d

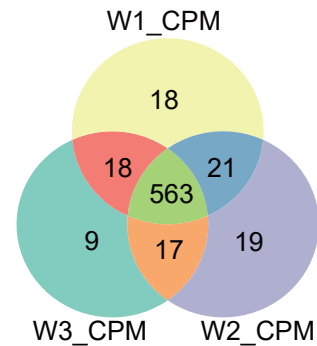

e

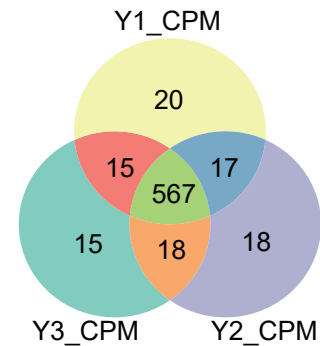

b

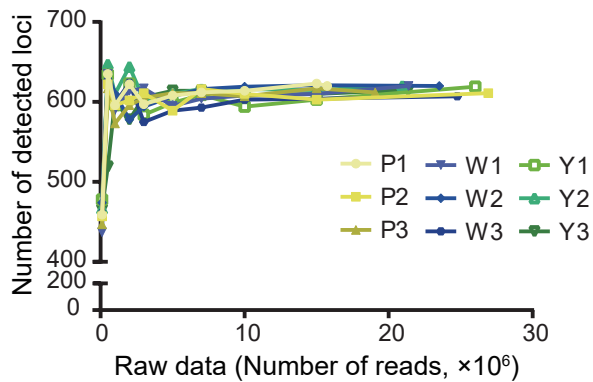

f

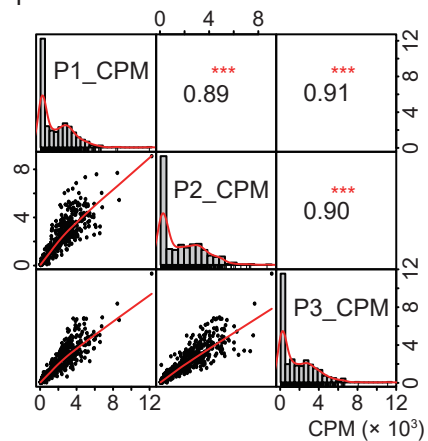

g

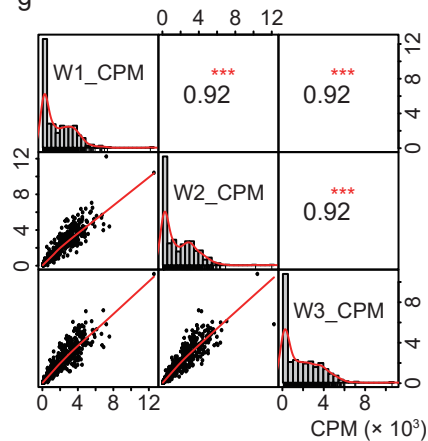

h

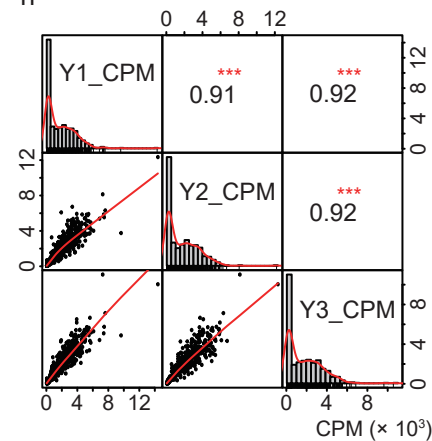

Supplement: Supplementary file 3 — Additional file 3: Fig. S2. Identification of HERV-K (HML-2) loci after filtering using a CPM ≥ 50. (a) Venn diagram exhibiting the interrelationship among the detected loci of different individuals. There are still 712 loci detectable after the filter. (b) Linear graph exhibiting the different number of raw reads that were extracted to calculate the number of detectable loci. (c-e) Venn diagram exhibiting the interrelationship among the detected loci of experimental replicates of different individuals. Within the 3 replicates of “P”, 566 loci could be detected in all the repeats, while in “W” and “Y”, there were 563 and 567 loci. (f–h) Correlation analysis of the relative abundance (CPM values) of the same loci in different samples (as in Fig. 3f–h). [file 12977_2020_519_MOESM3_ESM.pdf]
