# Supplementary material for: Identification of the distribution of human endogenous retroviruses K (HML-2) by PCR-based target enrichment sequencing
Source: Retrovirology. 2020 May 6;17:10. doi: 10.1186/s12977-020-00519-z (PMC7201656; doi:10.1186/s12977-020-00519-z)
Supplement: Supplementary file 4 — Additional file 4: Fig. S3. Phylogenetic analysis of all LTR nucleotide sequences of HERV-K (HML-2) detected by PTESHK. Phylogenetic analysis of all HERV-K (HML-2) possessing LTR sequences (provirus insertions that contained two LTRs only the 5′LTR was selected for alignment, if the 5′LTR was truncated, the 3′LTR was used). The tree was constructed by the neighbor-joining method using 5,000 bootstraps and the pair-wise deletion option. The NJ tree was well-clustered and annotated by the estimated age of every locus, the subtype of LTR (LTR5_Hs, LTR5A, and LTR5B), polymorphic insertion loci, the ability to be detected by PTESHK, and the CPM value. The results show good detection of known HERV-K (HML-2) loci, especially the LTR5_Hs type, which clustered within the longest internal branches and shortest terminal branches. [file 12977_2020_519_MOESM4_ESM.pdf]

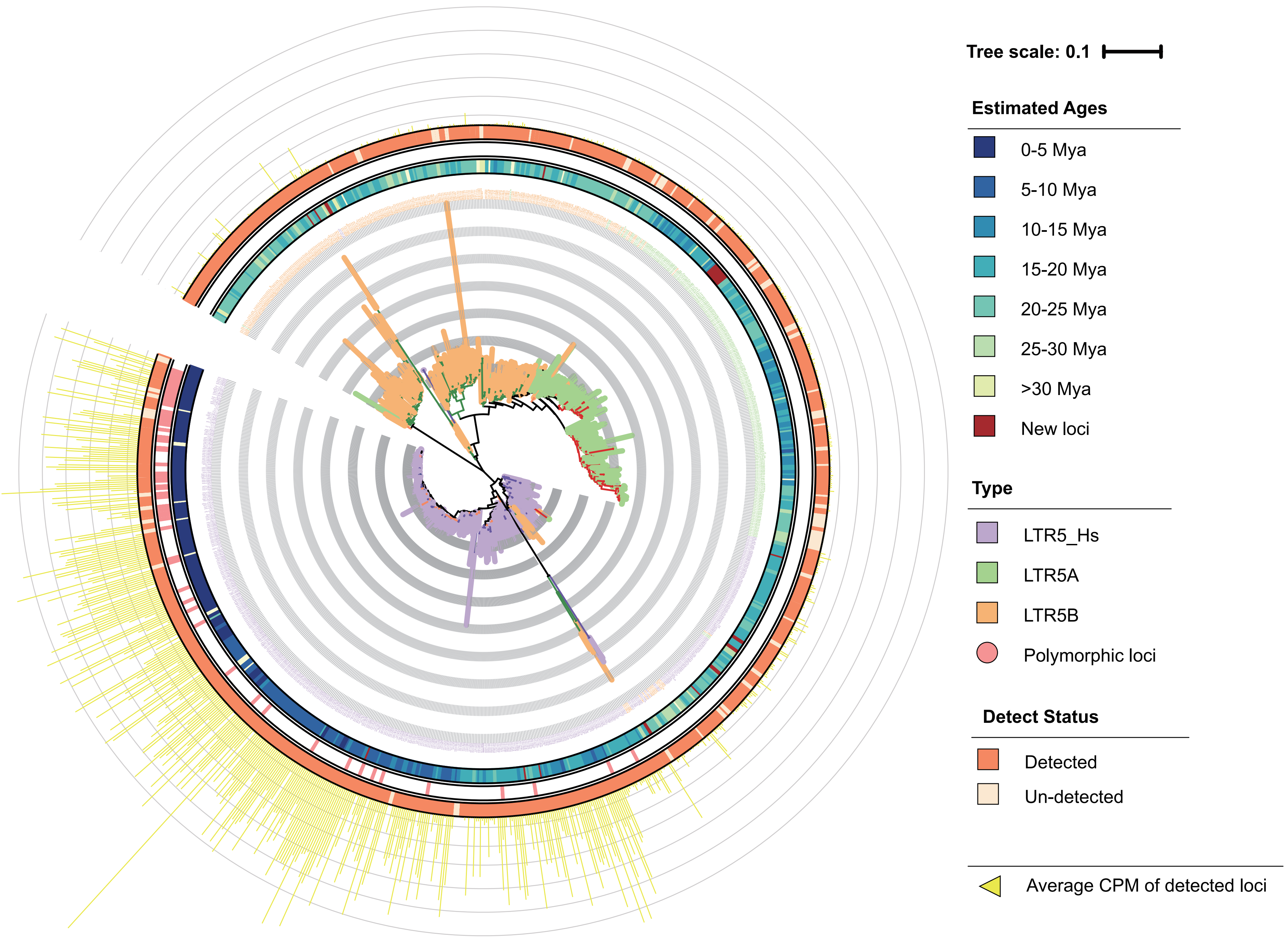

Tree scale: 0.1
Estimated Ages
0-5 Mya
5-10 Mya
10-15 Mya
15-20 Mya
20-25 Mya
25-30 Mya
>30 Mya
New loci
Type
LTR5_Hs
LTR5A
LTR5B
Polymorphic loci
Detect Status
Detected
Un-detected
Average CPM of detected loci
