# Supplementary material for: Identification of the distribution of human endogenous retroviruses K (HML-2) by PCR-based target enrichment sequencing
Source: Retrovirology. 2020 May 6;17:10. doi: 10.1186/s12977-020-00519-z (PMC7201656; doi:10.1186/s12977-020-00519-z)
Supplement: Supplementary file 5 — Additional file 5: Table S2. Nucleotide sequences for PCR verification of polymorphic loci. [file 12977_2020_519_MOESM5_ESM.doc]

Table S3. Nucleotide sequences for PCR verification of polymorphic loci

| Primary PCR | Sequence (5’-3’) | Nested PCR | Sequence (5’-3’) | Application |
| --- | --- | --- | --- | --- |
| N6_F1 | ATGGGATATTTGTCCATTTG | N6_F2 | CCTTGAGCAGTGGTTTGTAA | Chr6: 32643459-32643464 (+) |
| N6_R1 | TCACTTATGACAAGCCCACA | N6_R2 | ATAAGGAACTTCAGCAGTCT |
| LTR651_F1 | GAAGTCACGCTGAACATTAT | LTR651_F2 | TAAATGGTGCTGGAACGACA | Chr14: 20552744-20553715(+) |
| LTR651_R1 | ACATACTAAGACCCTCGTTC | LTR651_R2 | CACCGTGGAATGGCTAAATC |
| LTR585_F1 | GCGGGTTTGTCAGAGGTTCT | LTR585_F2 | GGACGAAGACCGATCTAAAC | Chr11:71875416-71876385(+) |
| LTR585_R1 | CAGAGGCAGATGATAACAGG | LTR585_R2 | TTGAACCAGGGAGTCGGAGG |
| LTR435_F1 | ATGATGGCGAGGTTGTGAAG | LTR435_F2 | CTGAGCAAACTACCGCAAGG | Chr8:18651457-18652426(+) |
| LTR435_R1 | AGGAATGAGGCACTGAGAAT | LTR435_R2 | ATACTTTACCCTTGACATACTC |
| LTR402_F1 | TGTGAAGTGAAATAAGCCAAAC | LTR402_F2 | TTTTCTACACTCGGCACCTC | Chr7:158029481-158030450 |
| LTR402_R1 | GGAGCGTTGGCTTGTGGACT | LTR402_R2 | CTTGACCGAGCAAACACCAG |
| LTR330_F1 | AATCCCAGAGGCGGAGGTTG | LTR330_F2 | CAGGAAGGGCAAGGTTATGT | Chr6:32624901-32625860(-) |
| LTR330_R1 | CTATTTGCTGCTATGAGGAT | LTR330_R2 | TGCCCTTGTTGTCTTTCACTA |
| 19q12_F1 | CACTCTTACAGTTGGGCACT | 19q12_F2 | TCGCTCAGGTGGGTGCTCTA | Chr19:29855781-29855787(-) |
| 19q12_R1 | ATCTGATGAAGTCACGCAAA | 19q12_R2 | AACTCCCGAAACACTACACC |
| 6p21.32b_F1 | CAGGCTATGTGAAGGTGGGA | 6p21.32b_F2 | AGGCGGACTTGAGAAGAGGA | Chr6:32505702-32505708(+) |
| 6p21.32b_R1 | ATCAGCAGCCATCAGGGAAA | 6p21.32b_R2 | ACAATGGTAAATGCTGGTGA |
| Using 5LTR2 as one of the nested PCR primers | | | | |
| LTR617_F1 | AACAACTGTGCAGCCCAACT | LTR617_F2 | CCATGTCCTATGACCGCTAT | Chr12:55727213-55728183(+) |
| LTR617_R1 | AGCCGAGATCGTGACACTGT |  |  |
| 4p16c_F1 | AGCCATCACCTACCACTCCA |  |  | Chr4:9603239-9603244(-) |
| 4p16c_R1 | CCTGGCTGGAACGAATGGTA | 4p16c_R2 | CGCTATGGCTCGCAGACAAC |
| 1p13.2_F1 | ACTTGCTGGGTGCTTGCTAA |  |  | Chr1:111802591-111802597(-) |
| 1p13.2_R1 | ATTTGCGACAATGTGGATGG | 1p13.2_R2 | TACTATCGTGGGACACTTCA |
| 19p12b_F1 | CCAATCCTCAACCCTAACTG |  |  | Chr19:21841535-21841541(-) |
| 19p12b_R1 | AGGGCATTAGTAAGTTGGTTT | 19p12b_R2 | TCAACAGGACGAGACAACTA |
| Primers failed to amplify | |  |  |  |
| 19p12d_F1 | TGCCAAGACAATCCTAAACA | 19p12d_F2 | TCATCAGAGTGAACAGGCAACC | Chr19:22414378- 22414383(-) |
| 19p12d_R1 | CACTATGCGACCTGAACTGC | 19p12d_R2 | TATGGCCTCATTTACAGACG |
| 6p21.32a_F1 | CTGCACCCATTAACTCGTCA | 6p21.32a_F2 | TTTGAATGTATGCGTGTCTT | Chr6:32648035-32648040(+) |
| 6p21.32a_R1 | TCTTCTGTCCCTTGCTACTG | 6p21.32a_R2 | GTGGTTGGCATAGTAGTGAT |
