# Supplementary material for: Identification of the distribution of human endogenous retroviruses K (HML-2) by PCR-based target enrichment sequencing
Source: Retrovirology. 2020 May 6;17:10. doi: 10.1186/s12977-020-00519-z (PMC7201656; doi:10.1186/s12977-020-00519-z)
Supplement: Supplementary file 6 — Additional file 6: Fig. S4. Verification of polymorphic loci. Selected polymorphic loci detected by PTESHK were verified using specific primers (Additional file 5: Table S2) and separated on a 1.5% agarose gel. Primer pairs F1/R1 were used for the primary PCR performed as follows: 95 °C for 3 min; 30 cycles of 95 °C for 30 s, 52 °C for 30 s, 72 °C for 1 min per kb of the product, and a final extension step at 72 °C for 10 min. Primer pairs F2/R2 were used for the nested PCR, except 4 loci using 5LTR2 as one of the nested PCR primers (Additional file 5: Table S2). The PCR procedure was performed as follows: 95 °C for 3 min; 6 cycles of 95 °C for 30 s, 60 °C for 30 s, decreasing of 1 °C every cycle, 72 °C for 1 min per kb of the product; 30 cycles of 95 °C for 30 s, 58 °C for 30 s, 72 °C for 1 min per kb of the product, and a final extension step at 72 °C for 10 min. Among all 14 polymorphic loci, 12 loci could be verified, except for 12p12d and 6p21.32a, which may be caused by either their location in a repeat element or a provirus integration where the length of the products were too long to amplify. For 6p21.32b and 19p12b, the results were partly confirmed, as 6p21.32b of Y and 19p12b of W were not amplified. This may be caused by the difference in DNA or experimental error. [file 12977_2020_519_MOESM6_ESM.pdf]

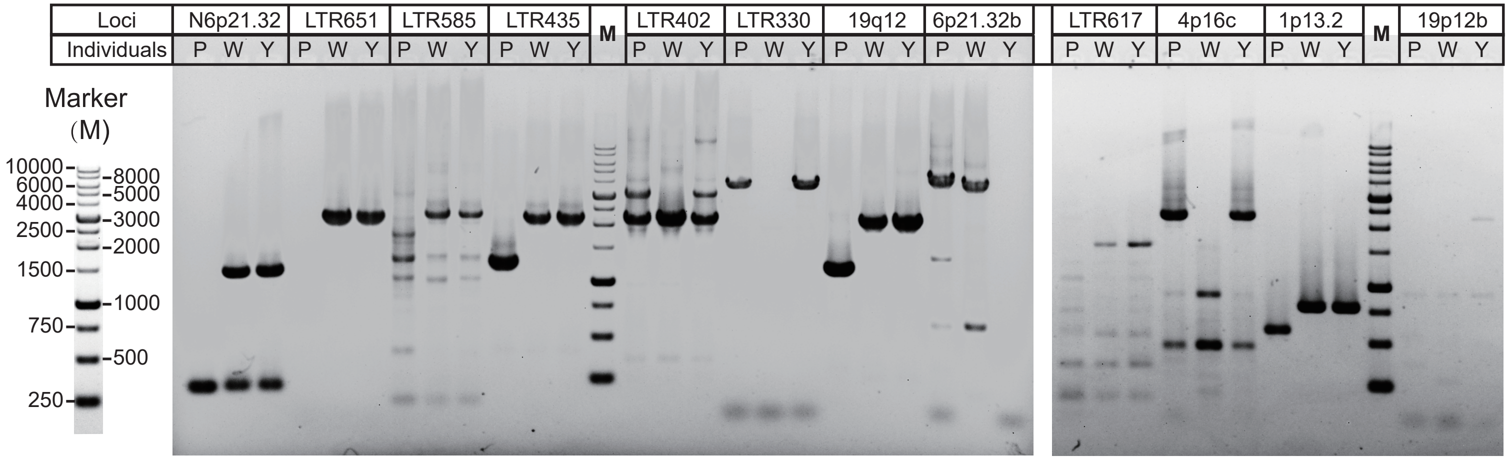

Loci
N6p21.32
LTR651
LTR585
LTR435
M
LTR402
LTR330
19q12
6p21.32b
LTR617
4p16c
1p13.2
M
19p12b
Individuals
P W Y
Marker (M)
10000
6000
4000
3000
2500
2000
1500
1000
750
500
250
8000
5000
