# Supplementary material for: Identification of the distribution of human endogenous retroviruses K (HML-2) by PCR-based target enrichment sequencing
Source: Retrovirology. 2020 May 6;17:10. doi: 10.1186/s12977-020-00519-z (PMC7201656; doi:10.1186/s12977-020-00519-z)
Supplement: Supplementary file 8 — Additional file 8: Table S4. Nucleotide sequences for NGS library construction. [file 12977_2020_519_MOESM8_ESM.doc]

Table S4. Nucleotide sequences for NGS library construction

| Primers | Sequence (5’ – 3’) | Application |
| --- | --- | --- |
| For NGS library construction amplifying all integration loci | | |
| KNG-A1 | GTGGCGGCCAGTATTCGTAGGAGGGCGCGTAGCATAGAACGT | GAPS linker |
| KNG-A2 | pCGTTCTATGCTAC |
| RBX4 | GTGGCGGCCAGTATTC | Primary PCR |
| 5LTR1 | ACAGATGCCTTCCTCTTRTC |
| 3LTR1 | GTCTGTGCTGAGGWGGATTAG |
| RBY1 | GAGGGCGCGTAGCATAGAAC | Nested PCR |
| 5LTR2 | TCYTGCACMGCCCTWRATCC |
| 3LTR2 | ACTRAGGGAACTCAGAGRCYGG |
